# Chemopreventive effect of a milk whey by-product derived from Buffalo (*Bubalus bubalis*) in protecting from colorectal carcinogenesis

**DOI:** 10.1186/s12964-023-01271-5

**Published:** 2023-09-20

**Authors:** Nunzio Antonio Cacciola, Tommaso Venneri, Angela Salzano, Nunzia D’Onofrio, Manuela Martano, Anella Saggese, Francesco Vinale, Gianluca Neglia, Ciro Campanile, Loredana Baccigalupi, Paola Maiolino, Mariarosaria Cuozzo, Roberto Russo, Maria Luisa Balestrieri, Michael John D’Occhio, Ezio Ricca, Francesca Borrelli, Giuseppe Campanile

**Affiliations:** 1 Department of Veterinary Medicine and Animal Production, University of Naples Federico II, Via F. Delpino, 1, Naples, 80137 Italy; 2 Department of Pharmacy, School of Medicine and Surgery, University of Naples Federico II, Via D. Montesano, 49, Naples, 80131 Italy; 3 Department of Precision Medicine, University of Campania Luigi Vanvitelli, Via L. De Crecchio, 7, Naples, 80138 Italy; 4 Department of Biology, University of Naples Federico II, Via V. Cupa Cintia, 21, Naples, 80126 Italy; 5 Institute of Genetics and Biophysics “A. Buzzati-Traverso”, National Research Council (CNR-IGB), Via P. Castellino 111, Naples, 80131 Italy; 6 Department of Molecular Medicine and Medical Biotechnology, University of Naples Federico II, Via S. Pansini, 5, Naples, 80131 Italy; 7 School of Life and Environmental Sciences, Faculty of Science, The University of Sydney, New South Wales, 2006 Australia

**Keywords:** Colorectal cancer, Delactosed milk whey by-product, Microbiota, Metabolome

## Abstract

**Background:**

Several studies show that natural foods are a source of compounds with anticancer properties that affect the gut microbiota and its metabolites. In the present study, we investigate the effect of a delactosed buffalo milk whey by-product (DMW) on colorectal carcinogenesis.

**Methods:**

The effect of DMW on colorectal carcinoma (CRC) was investigated in the established mouse model of azoxymethane (AOM)-induced colon carcinoma, which closely resembles the human clinical condition of CRC. The effect of DMW on CRC immortalized cell lines was also evaluated to further identify the antineoplastic mechanism of action.

**Results:**

Pretreatment of AOM-treated mice with DMW significantly (*P* < 0.05) reduced the percentage of mice bearing both aberrant crypt foci with more than four crypts (which are early precancerous lesions that progress to CRC) and tumors. In addition, DMW completely counteracted the effect of AOM on protein expression of caspase-9, cleaved caspase-3 and poly ADP-ribose polymerase in colonic tissue. Administration of DMW alone (i.e. without AOM) resulted in changes in the composition of the gut microbiota, leading to enrichment or depletion of genera associated with health and disease, respectively. DMW was also able to restore AOM-induced changes in specific genera of the gut microbiota. Specifically, DMW reduced the genera *Atopobiaceae*, *Ruminococcus 1* and *Lachnospiraceae* XPB1014 and increased the genera *Parabacteroides* and *Candidatus Saccharimonas*, which were increased and reduced, respectively, by AOM. Blood levels of butyric acid and cancer diagnostic markers (5-methylcytidine and glycerophosphocholine), which were increased by AOM treatment, were reduced by DMW. Furthermore, DMW exerted cytotoxic effects on two human CRC cell lines (HCT116 and HT29) and these effects were associated with the induction of apoptotic signaling.

**Conclusions:**

Our results suggest that DMW exerts chemopreventive effects and restores the gut microbiota in AOM-induced CRC, and induces cytotoxic effect on CRC cells. DMW could be an important dietary supplement to support a healthy gut microbiota and reduce the prevalence of CRC in humans.

Video Abstract

**Supplementary Information:**

The online version contains supplementary material available at 10.1186/s12964-023-01271-5.

## Background

Colorectal cancer (CRC) is the third most common cancer worldwide and the second leading cause of cancer-related deaths [1]. The prevalence of CRC has been steadily decreasing in people over the age of fifty [2–4] but at the same time it has been increasing in younger age groups [5, 6]. The American Cancer Society now recommends screening from age forty-five for people at average-risk [7]. However, CRC develops slowly over 10–15 years and only a small percentage of CRC are diagnosed early in young people [6, 8]. Risk factors for CRC include family background, diet, obesity, smoking, alcohol and gut microbiota dysbiosis [9–14]. The gut microbiota contributes to the intestinal metabolome, and alterations in the metabolome have been associated with CRC in both animal models [13] and humans [14]. Diet influences the gastrointestinal microbiota as well as the gut and blood metabolome, and longer-term management of diet has the potential to be an important adjunct to other intervention that reduce the risk profile for developing CRC.

Natural products have been shown to be as a valuable source of factors and metabolites with anti-cancer properties. The protective effect of milk whey proteins on colon carcinogenesis has been known for about thirty-five years [15]. More recently, we have shown that non-protein metabolites in buffalo milk whey by-product have antiproliferative and pro-apoptotic effects on human cancer cells in vitro, including colorectal cancer cells [16]. Of particular note is the potent effect of γ-butyrobetaine, δ-valerobetaine, and other carnitine precursors on cancer cells [17, 18]. We have also performed a first in vivo study using the HCT116 xenograft mouse model for colorectal cancer [19]. Buffalo milk whey by-product upregulated apoptotic signaling pathways in xenograft tissue, but there was no apparent effect on xenograft tumor volume [19]. The aim of the present study was to extend our previous work by using the well-established mouse model of azoxymethane (AOM)-induced colon cancer, which mimics the development of sporadic CRC [20, 21] and also induces alterations in the gut microbiota and metabolome typical of CRC [22–25]. The effects of DMW on two human colon adenocarcinoma cell lines (HCT116 and HT29) were also studied to confirm the identified in vivo mechanism of action.

## Material and methods

### Delactosed and defatted buffalo milk whey by-product

Milk from Mediterranean Italian dairy buffaloes (*Bubalus bubalis*) was obtained from 30 commercial buffalo farms in the Caserta region of southern Italy. The milk was subjected to enzymatic hydrolysis with the enzyme lactase (neutral pH and at 25 °C) to obtain delactosed milk. Then the whey obtained by adding curd to the milk was used to make ricotta. Finally, the whey residue obtained from the ricotta was subjected to reverse osmosis to obtain a concentrated solution of 60% delactosed milk whey by-product (DMW).

For the in vitro experiments, the DMW was defatted to obtain DMW without fat (DMW/WF). Briefly, the DMW was centrifuged three times at 23,000 g (4°C for 30 min) and then centrifuged at 23,000 g for another 1 h [26]. The solution was aliquoted and frozen until use. Before use, the DMW/WF was added to the complete cell culture medium and the solution was finally passed through a 0.22 µm Millipore filter (Merck Millipore, Milan, Italy).

### Milk whey by-product metabolomic profiling

The metabolomic profile of DMW has been reported previously [19]. DMW/WF metabolomic analysis was performed under the same experimental conditions as reported by Cacciola et. al [19]. Quantification of δ-valerobetaine (δ-VB) content was performed by HPLC-ESI MS/MS [27].

### In vivo induction of colon cancer

#### Animals and treatments

Male C57BL/6 mice (6–8 weeks old, Charles River Laboratories, Calco, Lecco, Italy) were housed in polycarbonate cages (6 mice per cage) and used after a 1 week acclimation period (temperature 23 ± 2 °C; humidity 60%, 12-h light/dark cycle with light on at 07:00 AM). The mice had free access to water and a standard rodent diet (Mucedola S.r.L, Milan, Italy).

All experimental procedures were assessed and approved by the Institutional Animal Ethics Committee for the use of experimental animals and complied with the guidelines for the safe use and care of experimental animals according to the Italian D.L. no. 116 of 27 January 1992 and associated guidelines in the European Communities Council (86/609/ECC and 2010/63/UE).

**Fig. 1:**
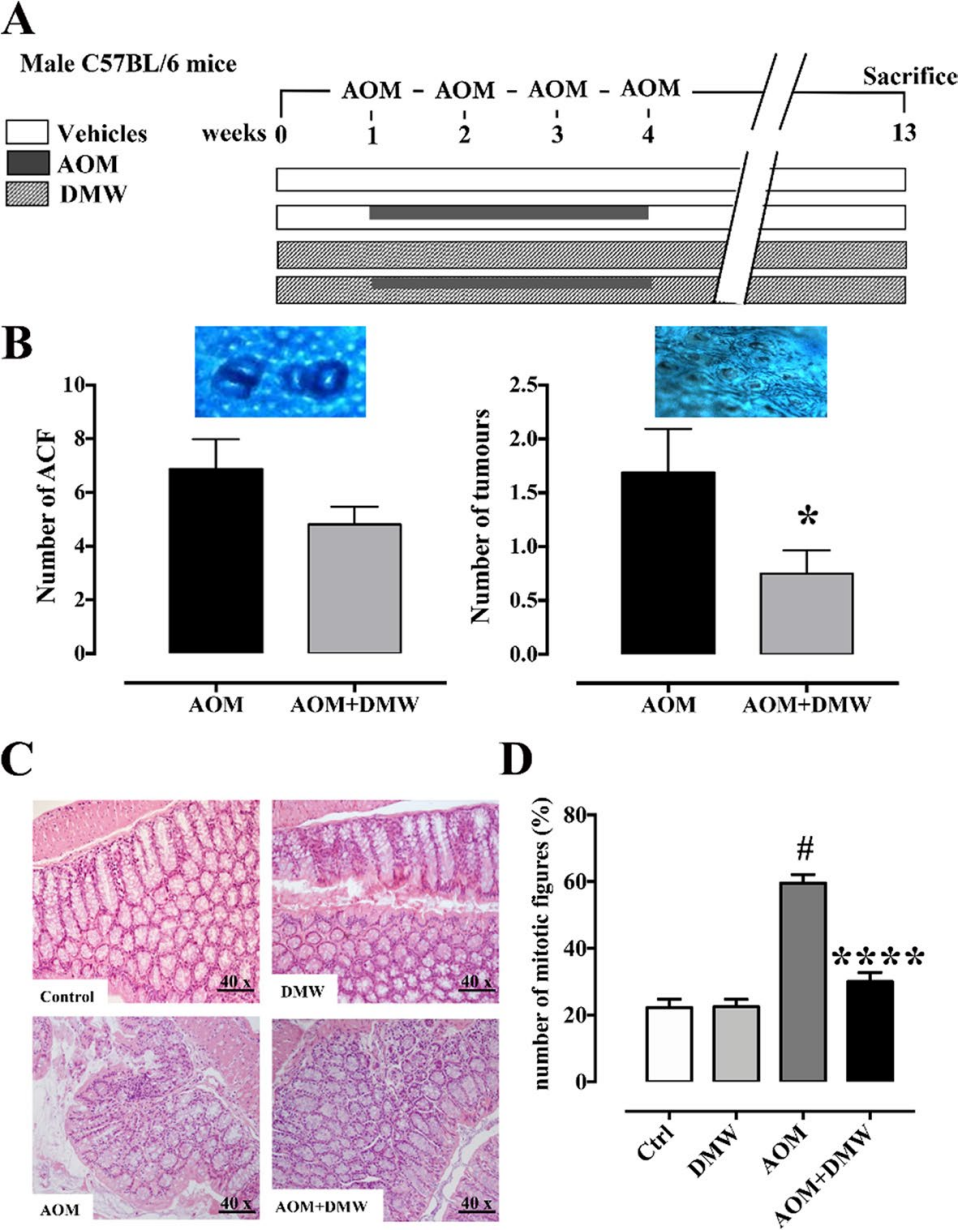
Oral administration of delactosed milk whey by-product (DMW) from Mediterranean Italian dairy buffaloes prevents carcinogenesis development in the mouse model of azoxymethane (AOM)-induced colon cancer. **A** Experimental design related to the AOM-induced tumor model; **B** Number of aberrant crypt foci (ACF) and tumors induced in the mouse colon by AOM (inserts above the graphs show an example of ACF and tumor). AOM (40 mg/kg in total, intraperitoneally) was administered, at the single dose of 10 mg/kg, at the beginning of the first, second, third and fourth week. DMW was given (by oral gavage), at the 10 ml/kg dose, three times a week for the whole duration of the experiment starting 1 week before the first administration of AOM. Measurements were performed at the end of experiment (i.e., 13 weeks after the first injection of AOM). Data represent the mean ± SEM of 16 mice. *p < 0.05 *vs* AOM alone. **C** Histological features of colon from control and treated mice. Control and DMW mice colon showed normal morphology; AOM treated mice colon showed adenomatous polyp with signs of dysplasia; AOM plus DMW treated mice colon showed polypoid hyperplasia (H-E; Original magnification × 40; scale bar 50 µm). **D** The number of mitotic figures (%) was counted in colon sample (obtained from control, DMW, AOM and AOM plus DMW treated mice) considering 10 randomly selected high-power fields (HPF; 40X objective 10X ocular). Data represent the mean ± SEM of 4/5 mice. #*p* < 0.0001 *vs* control (Ctrl), ****p < 0.0001 *vs* AOM alone

The mice were randomly divided into four groups: group 1 (control, Ctrl, *n* = 16), intraperitoneal (i.p.) injection of vehicle; group 2 (DMW, *n* = 16), oral administration of DMW (10 ml/kg) three times a week for the duration of the experiment; group 3 (AOM, *n* = 16), i.p. injection of AOM (10 mg/kg; Merck Millipore) at the beginning of the 1st, 2nd, 3rd and 4th week [27]; group 4 (AOM + DMW, *n* = 16), AOM and DMW administered as in groups 2 and 3 (Fig. 1A). AOM treatment has been shown to induce a significant number of aberrant crypt foci (ACF) and tumors [28]. All animals were euthanized by CO_2_ asphyxiation thirteen weeks after the first injection of AOM.

#### Preneoplastic lesions and tumors

Aberrant crypt foci (ACF) and tumors were evaluated in mice colons as previously described [29]. Briefly, the colons were quickly removed after euthanasia, washed, opened longitudinally, laid flat and fixed with 10% buffered formaldehyde. After staining with 0.2% methylene blue in saline, the colons were examined for the detection of ACF and tumors using a light microscope at 20 × magnification (Leica Microsystems, Milan, Italy) by two independent investigators in a blinded fashion.

#### Histological evaluation

After sacrifice, colon segments (1.5 cm) were fixed in 10% formaldehyde for 24 h. The samples were then embedded in paraffin and cut into 4 µm sections. For histological evaluation, the samples were stained with hematoxylin and eosin (H-E) (Carlo Erba Reagents SAS, Val de Reuil, France). Histological evaluation and counting of mitotic figures (MF) were performed with a microscope (E-400; Nikon Eclipse, Tokyo, Japan) by two independent observers blinded to the treatments. MF were counted from 10 randomly selected high-power fields (HPF; 40 × objective 10 × ocular) for each colon sample, as previously described [30].

#### Confocal laser scanning microscopy

Immunofluorescence analysis on deparaffinized sections was performed according to the manufacturer’s protocol using a confocal laser-scanning microscope (LSM 700, Zeiss) with a Plan-Apochromat X63 (NA1.4) oil immersion objective. Briefly, antigen retrieval buffer (10 mM sodium citrate, 0.05% Tween 20, pH 6.0) was added to deparaffinized and rehydrated sections and boiled in the microwave for 20 min. After blocking with 5% fetal bovine serum (FBS) for 1 h, immunofluorescence staining was performed with a specific primary antibody against PARP (1:500, ab194586, abcam), followed by incubation with anti-rabbit secondary antibody Alexa Fluor 633. Phalloidin-iFluor 488 reagent (1:1000, ab176753, abcam) was used to detect F-actin filaments while staining of the nucleus was performed using 2.5 µg/ml 4',6-diamidino-2-phenylindole (DAPI, Sigma Aldrich). To remove the nonspecific background noise, sections were incubated with Vector TrueVIEW autofluorescence quenching kit (SP-8400, Vector Laboratories) before mounting. Fluorescence intensity was estimated using ImageJ software and expressed as arbitrary fluorescence units (AFU).

#### Microbiota identification by 16S rRNA sequencing

Fecal samples (300 mg) from mice were collected at time 0 (before the start of treatment, week 0) and after 13 weeks of treatment (week 13) and total DNA was extracted using the QIAamp DNA Stool Mini Kit (QIAGEN) according to the manufacturer’s instructions. Partial 16S rRNA gene sequences were amplified using the primer pair Probio_Uni and Probio_Rev, targeting the V3 region of the 16S rRNA gene [31]. Amplicon checks were performed as previously described [31]. Sequencing of the 16S rRNA gene was performed using a MiSeq (Illumina) in the GenProbio srl DNA sequencing facility (www.genprobio.com) following the protocol described previously [30]. After sequencing and demultiplexing, the reads obtained from each sample were filtered to remove low-quality and polyclonal sequences. All quality-approved, trimmed and filtered data were exported as.fastq files. The.fastq files were processed using a script based on the QIIME software suite [32]. Paired-end reads pairs were assembled to reconstruct the complete Probio_Uni / Probio_Rev amplicons. After quality control, sequences with a length between 140 and 400 bp and mean sequence quality score > 20 were retained. Sequences with homopolymers > 7 bp and mismatched primers were omitted.

To calculate downstream diversity measures (alpha and beta diversity indices, Unifrac analysis), 16S rRNA Amplicon Sequence Variants (ASVs) were defined at ≥ 100% sequence homology using DADA2 [33] and ASVs that did not encompass at least 2 sequences from the same sample were removed. All reads were classified to the lowest possible taxonomic rank using QIIME2 [32, 34] and the SILVA database v. 132 as the reference dataset [35]. The biodiversity of the samples (alpha-diversity) was calculated using the Chao1 and Shannon indexes. The similarities between the samples (beta-diversity) were calculated using the weighted uniFrac [36]. The range of similarities is calculated between the values 0 and 1. PCoA representations of beta-diversity were performed with QIIME2 [33, 35].

#### Measurement of blood short chain fatty acid (SCFA)

At the end of the experiments, blood was taken from each animals by cardiac puncture and serum was obtained by centrifugation (4 °C, 10 min, 800 g). The serum was analyzed by gas-chromatography mass spectrometry (GC—7890A, Agilent Technologies; MS—5977A MSD, Agilent Technologies). In brief, serum samples were acidified with 20 μl phosphoric acid (H_3_PO_4_) 85% (w/v), vortexed for 5 min and incubated on ice for 5 min. The acidified samples were extracted by adding diethyl ether (DE) for acetic and propionic acids (1:1, v/v), and ethyl acetate (EA) for butyric acid (1:1, v/v), vortexed for 5 min and then centrifuged at 12,000 × g for 20 min at RT. Anhydrous sodium sulphate was added to the organic extract (containing the SCFAs) to remove the residual water and the samples were transferred to a new glass-tube for GC–MS analysis. A standard curve (0.1–3 µg/ml) was generated for each SCFA at the beginning of the run. A blank solvent (DE or ethyl acetate) was injected between each sample to ensure that no memory effects occurred. The GC was programmed to achieve the following run parameters: initial temperature of 90 °C, hold of 2 min, ramp of 2 °C/min up to a temperature of 100 °C, hold of 10 min, ramp of 5 °C/min up to a final temperature of 110 °C for a total run time of 21 min.

#### Untargeted metabolomic analysis of blood

The water residues of the serum after extraction with ethyl acetate and petroleum ether (see section Measurement of SCFA concentration in serum) were used for LC–MS Q-TOF analysis. The untargeted metabolomic study was performed using an Agilent HP 1260 Infinity Series liquid chromatography coupled to a Q-TOF mass spectrometer and equipped with a DAD system (Agilent Technologies, Santa Clara, CA, USA). The MS system was equipped with a Dual Electrospray Ionization (ESI) source which operated in positive mode. Chromatographic separation was performed using an Adamas C18-X-Bond column (50 mm X 4.6 mm, 3.5 µm, Sepachrom Srl) held at a constant temperature of 25 °C. Analyses were performed at a flow rate of 0.5 ml min-1, using a linear gradient system composed of 0.1% (v/v) formic acid in water (eluent A), and 0.1% (v/v) formic acid in acetonitrile (eluent B). The gradient program was set as follows: starting from 5 to 70% eluent B in 4 min, isocratic at 70% of eluent B from 4 to 5 min; from 70 to 80% of eluent B from 5 to 8 min and from 80 to 100% eluent B from 8 to 10 min; lowering to starting condition (5% eluent B) from 10 to 15 min to achive the equilibration after 1 min. The injection volume was 10 µL. UV spectra were recorded from DAD every 0.4 s from 190 to 750 nm, with a resolution of 2 nm. The capillary was maintained at 2000 V, fragmentor voltage at 180 V, cone 1 (skimmer 1) at 45 V, Oct RFV at 750 V. The gas flow rate was set to 11 L min-1, at 350 °C, and the nebulizer was set at 45 psig. Mass spectra were recorded within the m/z range 100–1700 as centroid spectra, with three scans per second. To perform real time mass-lock correction, a solution consisting of purine (C_5_H_4_N_4_, m/z 121.050873, 10 µmol L-1), and hexakis (1H,1H,3H-tetrafluoropentoxy)-phosphazene (C_18_H_18_O_6_N_3_P_3_F_24_, m/z 922.009798, 2 µmol L-1) was constantly infused with an Isocratic pump (1260 Infinity Series, Agilent Technologies) at a flow rate of 0.06 ml min-1. All MS and HPLC parameters were determined using Agilent MassHunter Data Acquisition Software, rev. B.05.01.

### In vitro cytotoxicity

#### Cell lines and cell cultures

Two human colon adenocarcinoma cell lines, HCT116 and HT29 (ATCC from LGC Standards, Sesto San Giovanni, Milan, Italy), and a healthy human colonic epithelial cell line, HCEC (Fondazione Callerio Onlus, Trieste, Italy), were used. HCT116 and HT29 cells were cultured in McCoy's 5A medium (Euroclone, Milan, Italy) containing 10% FBS (Microgem, Naples, Italy), 2 mM L-glutamine (Microgem), 100 U/ml penicillin and 100 μg/ml streptomycin (Microgem). HCEC cells were maintained in Dulbecco’s modified Eagle’s medium (Merck Millipore, Milan, Italy) supplemented with 10% FBS, 100 U/ml penicillin, 100 μg/ml streptomycin, 20 mM HEPES [4-(2-hydroxyethyl)-1-piperazineethanesulfonic acid] (Merck Millipore), 2 mM L-glutamine (Merck Millipore), and 1 mM Na-pyruvate (Merck Millipore). The medium was changed every 48 h according to the manufacturer’s protocols. Cells from passage 9 to 20 tested for mycoplasma contamination were used.

#### Cell viability

The cell viability was determined using the MTT assay [28]. Briefly, cells were seeded in 96-well plates (at a density of 1 × 10^4^ cells *per* well for HCT116 and HCEC and 1.5 × 10^4^ for HT29) and allowed to adhere for 24 h. The cells were then exposed to increasing concentrations of DMW/WF from 2 to 20% v/v for 24 h. Cells were then incubated with 3-(4,5-dimethylthiazol-2-yl)-2,5-diphenyltetrazolium bromide (250 μg/ml, Merck Millipore) for 1 h at 37 °C, and the formation of formazan was measured at 570 nm using a microplate reader (BioTek™ Cytation™ 3, Winoosky, USA). Results are expressed as percentage of cell viability (*n* = 3 experiments with 3–6 replicates for each treatment). Morphological changes of cells, treated or not with DMW/WF (0.3–20% v/v) for 24 h, were analyzed by scanning and phase contrast photography with a Zoe™ Fluorescent Cell Imager (Biorad, Germany).

#### Cell apoptosis

Apoptosis was assessed by cytofluorimetric analysis using the Annexin V and propidium iodide (PI) apoptosis assay Kit (Dojindo Molecular Technologies Inc., Munich, Germany) as previously described [37]. Briefly, HCT116 and HCEC cells were seeded in six-well plates (4 × 10^5^ cells/well and 4.5 × 10^5^ cells/well for HCT116 and HCEC, respectively) and cultured in the absence or presence of DMW/WF (1–10% v/v) for 24 h. Subsequently, cells were harvested, washed twice with PBS, resuspended in Annexin V binding buffer and incubated with 5 μL Annexin V and 5 μL PI for 15 min at room temperature (RT). Finally, for HCT116 cells, apoptotic cells were analyzed using the BD FACSCanto II system and analysis performed using DIVA software (BD Biosciences, Milan, Italy). For HCEC cells, apoptotic cells were analyzed using the BriCyte E6 (Mindray, P.R. China). At least 10,000 events were recorded for each condition.

#### Cell and tissue lysate preparation

HCT116 cells were seeded in 6 cm^2^ Petri dishes at a density of 1 × 10^6^ cells and allowed to adhere for 24 h. Cells were then incubated with DMW/WF (6% or 10% v/v) for 0, 3, 6, 12, and 24 h. At each endpoint, cells were washed twice with ice-cold 1 × PBS, collected by scraping and lysed in RIPA buffer (Cell Signaling Technology, USA). After 30 s of sonication, lysates were centrifuged at 14,000 g (4 °C for 15 min), the supernatant was collected, and protein concentration was determined using the Bio-Rad DC protein assay (Bio-Rad, Munich, Germany). Total protein lysates from colon tissues were extracted as previously described [38]. In brief, colon tissues were washed with chilled 1 × PBS to remove all blood contaminants. The colon tissues were then dissected into small pieces, lysed in RIPA buffer using an Ultra Turrax™ device (Ika-Werke GmbH, Staufen, Germany) and sonicated for 30 s. Finally, the lysates were centrifuged at 14,000 g (4 °C for 15 min), the supernatant was collected and the protein concentration was determined using the Bio-Rad DC protein assay (Bio-Rad).

#### Western blot analysis

For Western blot analysis, colon tissue lysates (70 μg) were resolved on 4–20% SDS–polyacrylamide gels and then transferred to nitrocellulose membranes (Bio-rad, Germany) using the Trans-blot® Turbo ™ transfer system from Bio-rad. Similarly, cell lysates (40 µg) were resolved on 4–20% SDS–polyacrylamide and transferred to nitrocellulose membranes (Bio-rad, Germany) using the wet transfer method. After blotting, the membranes derived from the tissues and cells were blocked with 5% nonfat milk in Tris Buffered Saline (TBS) solution containing 20 mM Tris (pH 7.6), 137 mM NaCl with Tween® 20 detergent (TBS-T) for 1 h at RT. The membranes were then cut according to the standard bands position and probed with primary antibodies against cleaved caspase-3 (#9664 Cell Signaling Technology Inc., USA,1:1000, for in vivo and in vitro experiments), caspase-9 (#9508, Cell Signaling Technology Inc., 1:1000, for in vivo experiments), PARP (#9532, Cell Signaling Technology Inc., 1:1000, for in vivo experiments), cleaved caspase-9 (#20,750, Cell Signaling Technology Inc., 1:1000, for in vitro experiments), cleaved caspase-8 (#9496 Cell Signaling Technology Inc., 1:1000, for in vitro experiments), and cleaved PARP (#5625, Cell Signaling Technology Inc., 1:1000, for in vitro experiments), overnight at 4 °C on a rocker. β-Actin (sc-47778, Santa Cruz Biotechnology Inc., USA, 1:10, 000) or Vinculin (ab129002, Abcam, UK, 1:10, 000) were incubated for 1 h at RT. The membranes were then washed 3 times for 5 min with TBS-T buffer and incubated with horseradish peroxidase-conjugated secondary antibody (Cell Signaling Technology Inc., 1:2000) at RT for 1 h. The immunoreactive bands were detected on X-ray films using the enhanced chemiluminescent kit and quantified by densitometry using ImageJ software (National Institutes of Health, Bethesda, MD, USA).

### Statistical analyses

Statistical analyses were performed using GraphPad Prism 9.0 (GraphPad software, San Diego, CA, USA). Statistical significance was determined using either Student's t test for comparing a single treatment mean with a control mean or One-way analysis of variance (ANOVA) followed by Tukey’s multiple comparison test for analysis of multiple treatment means. Two-way ANOVA followed by the Bonferroni multiple comparisons post-test was used to compare different cumulative concentration effect curves. The DMW concentration that produced 50% inhibition of cell viability (EC_50_) or maximum inhibitory effect (E_max_) was calculated by non-linear regression analysis using the equation for a sigmoid concentration–response curve.

To determine the SCFA concentration in µg/ml using Graphpad Prism, the data were inserted in "XY" form, with the "X" field indicating the values of the straight concentration–response curve, while the "Y" field indicated the values of the area under the curve (AUC) with respect to the peaks obtained from the mass gas. The AUC values of the individual samples (obtained from the mass gas) were interpolated with the line X (concentration–response relationship) to determine the corresponding concentration.

For untargeted metabolomic analysis, raw data were analyzed using Mass Profile Professional 13.1.1 software (Agilent Technologies) and divided into the following groups: AOM + DMW, AOM, DMW and compared with the control group. In addition, the groups AOM + DMW and AOM were compared, with AOM serving as the control group. For statistical analysis all groups were subjected to One-way ANOVA (*p*-value < 0.05) and to fold change ≥ 2.0. Principal components analysis (PCA) were performed to compare metabolic profiles of serum samples. Statistically relevant compounds were putatively identified using an in-house serum metabolites database, a freely available electronic database, Human metabolome database (HMDB).

Analysis of fecal microbiota composition was performed using SPSS software 25 (www.ibm.com/software/it/analytics/spss/) and differences between experimental groups, including differences in abundance of bacterial genera, were determined by One-way or Two-way ANOVA analyses followed by Tukey’s or LSD (least significant difference) multiple comparisons tests. For all analyses *p* < 0.05 was considered significant. Results are presented as means ± SEM of *n* experiments.

## Results

### Milk whey by-product metabolome profiling

The metabolome profiling of milk whey by-product was obtained by HPLC–MS analysis. Mass spectra and chromatograms were used to putatively identify metabolites by comparison with public databases, data from the literature and, where possible, by comparison with authentic standards. The main constituents identified in DMW/WF are listed in Table 1. Eight metabolites were identified in DMW/WF samples, belonging to the classes amino acids, fatty acids, tricarboxylic acids, hydroxycoumarin derivatives, triterpenes, amino alcohols and O-glycoside. Compared to DMW, defatted DMW resulted in a loss of lipophilic compounds such as erucamide and butyryl-L-Carnitine [19]. To titrate the DMW/WF, we determined the δ-valerobetaine content to be 284 µM.

**Table 1:** Compounds identified in delactosed milk whey by-product without fat (DMW/WF) from Mediterranean Italian dairy buffaloes (*Bubalus bubalis*) by HPLC–MS analysis

RT (min)	Experimental *m/z* [M-H]^+^ (Da)	Theoretical Mass (Da)	Mass Error (ppm)	Molecular formula	Putatively identified compound	Metabolite class
0,697	198,9397	197,93,501	1,00469	C_4_H_7_BrO_2_S	3-Bromosulfolane	Triterpenoid
0,750	365,1044	364,094688	1,009712	C_21_H_16_ O_6_	Gerberinol	4-Hydroxycoumarin
0,812	203,0521	202,047738	1,004362	C_8_H_10_O_6_	Ethyl aconitate	Trycarboxylic acid
0,816	162,1119	161,105,193	1,006707	C_7_H_15_NO_3_	L-Carnitine	Amino acid
0,828	160,1325	159,125,929	1,006571	C_8_H_17_NO_2_	Δ-Valerobetaine	Straight chain fatty acids
0,843	204,1226	203,115,758	1,006842	C_9_H_17_NO_4_	Acetyl-L-carnitine	Amino acid
0,870	381,078	380,077718	1,000282	C_14_H_20_O_10_S	4-Methoxybenzyl O-(2-sulfoglucoside)	O-glycoside
5,959	230,2483	229,240,565	1,007735	C_14_H_31_NO	Xestoaminol C	1,2-Aminoalcohol

### In vivo induction of colon cancer

#### Effect of AOM and DMW on colon cancer

After 3 months of treatment, AOM triggered the appearance of aberrant crypt *foci* (ACF) and tumors (Fig. 1 B). Pretreatment of AOM mice with DMW decreased (*p* < 0.05) the percentage of mice bearing i) ACF containing more than four crypts, ii) tumors and iii) more than one tumor (Table 2). DMW also reduced (*P* < 0.05) the number of tumors (Fig. 1B); there was only a trend to reduce ACF formation (30% inhibition, *p* = 0.12). No formation of ACF and tumors was observed in control and DMW treated mice (data not shown).

**Table 2:** Effect of delactosed milk whey by-product (DMW; 10 ml/kg) from Mediterranean Italian dairy buffaloes (*Bubalus bubalis*) on the percentage of mice bearing aberrant crypt *foci* (ACF), ACF with more than four crypts, tumors, or more than one tumor, in the mouse model of azoxymethane (AOM)-induced colon cancer

**Treatment**	**N**	**Mice bearing**			
		**ACF**	**ACF > 4 crypts (%)**	**Tumors**	**Tumors > 1**
AOM	16	100	75	75	43.8
AOM + DMW	16	100	33.3*	56.3	12.5*

^*^p < 0.05 *vs* AOM alone (chi-square test)

#### Effect of AOM and DMW on colon histology

No histological changes were observed in the colonic mucosa of the control and DMW-treated mice. In these mice, the crypts were well organized, aligned and parallel, and characterized by a single layer of columnar and goblet cells (Fig. 1C). The colon tissues of the AOM-treated mice showed pathological changes including focal atypia, dysplasia and hyperplasia, as well as adenomatous polyps characterized by branched tubules in the *lamina propria* (Fig. 1C). In addition, the tissues were disorganized and covered by epithelium with nuclear pseudostratification and signs of dysplasia (basal cells disorganization, hypercromasia, loss of polarity) (Fig. 1C). In mice treated with AOM plus DMW, the colons had areas of polypoid hyperplasia, characterized by a simple tubular architecture with elongated and straight crypts with nuclear hypercromasia and maintenance of polarity (Fig. 1C). DMW-treated mice had a similar number of mitotic figures (MF) as control mice (Fig. 1D). AOM treatment increased (*p* < 0.0001) the number of MF, and this effect was reduced (*p* < 0.0001) by DMW (Fig. 1D).

### Effect of AOM and DMW on apoptotic pathway

**Fig. 2:**
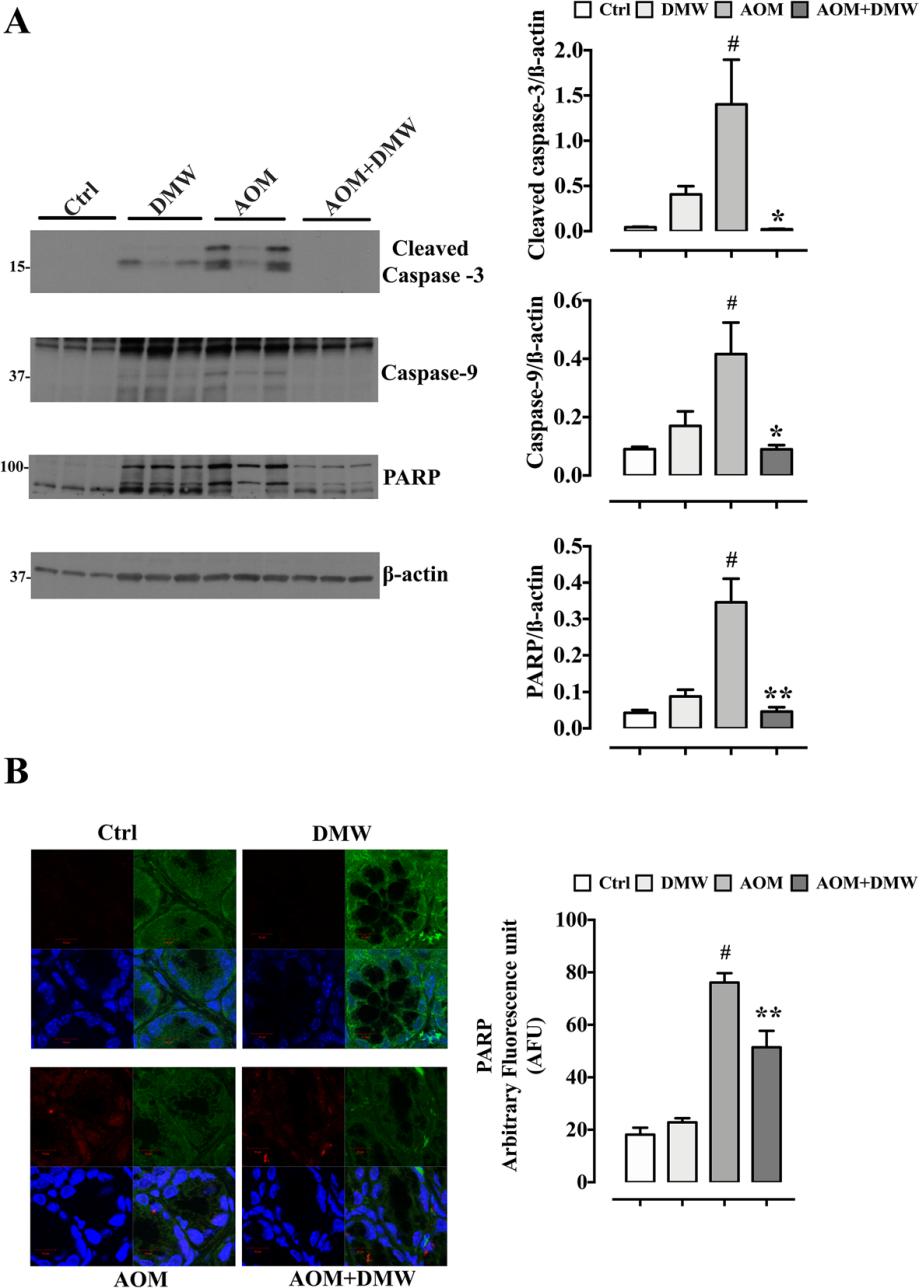
Oral administration of delactosed milk whey by-product (DMW) from Mediterranean Italian dairy buffaloes affects apoptosis-related proteins in the colon of azoxymethane (AOM)-treated mice. **A** Western blot analysis showing the protein expression levels of cleaved caspase-3, caspase-9 and poly (ADP) ribose polymerase (PARP) in colon of animals treated or not with AOM or/and DMW. AOM (40 mg/kg in total, intraperitoneally) was administered, at the single dose of 10 mg/kg, at the beginning of the first, second, third and fourth week. DMW was given (by oral gavage), at the 10 ml/kg dose, three times a week for the whole duration of the experiment starting 1 week before the first administration of AOM. Measurements were performed at the end of experiment (i.e., 13 weeks after the first injection of AOM). The right side of the panel shows the densitometry analysis of the above proteins. Signals were normalized on the housekeeping proteins β-actin. Data represent the mean ± SEM of three individual mice tissues. #*p* < 0.01 *vs* control (Ctrl, untreated mice), *p < 0.05, and **p < 0.01 *vs* AOM alone. **B** Representative immunofluorescence images performed on deparaffinized sections of colon from mice treated or not with AOM or/and DMW showing PARP fluorescence intensity expressed as arbitrary fluorescence units (AFU). Results are mean ± SEM of three independent experiments. #*p* < 0.0001 *vs* control (ctrl, untreated mice) and **p < 0.01 *vs* AOM alone

To investigate the mechanism responsible for the chemopreventive effects of DMW, we examined the protein expression of caspase-9, cleaved caspase-3, and PARP in colonic tissues of mice treated with AOM. Western blot analysis of three randomly selected samples from three individual mice showed increased protein expression levels of cleaved caspase-3, caspase-9, and PARP after AOM administration (*p* < 0.0001). Pretreatment with DMW completely reversed the effect of AOM on these protein markers (Fig. 2A). The effect of DMW on PARP protein expression was confirmed by immunofluorescence microscopy analysis. Quantification of PARP-positive cells showed a strong signal in the AOM group (*p* < 0.0001) which was partially counteracted by DMW treatment (Fig. 2B). DMW alone did not change any of the markers analyzed (Fig. 2).

### Effect of AOM and DMW on intestinal microbiota

**Fig. 3:**
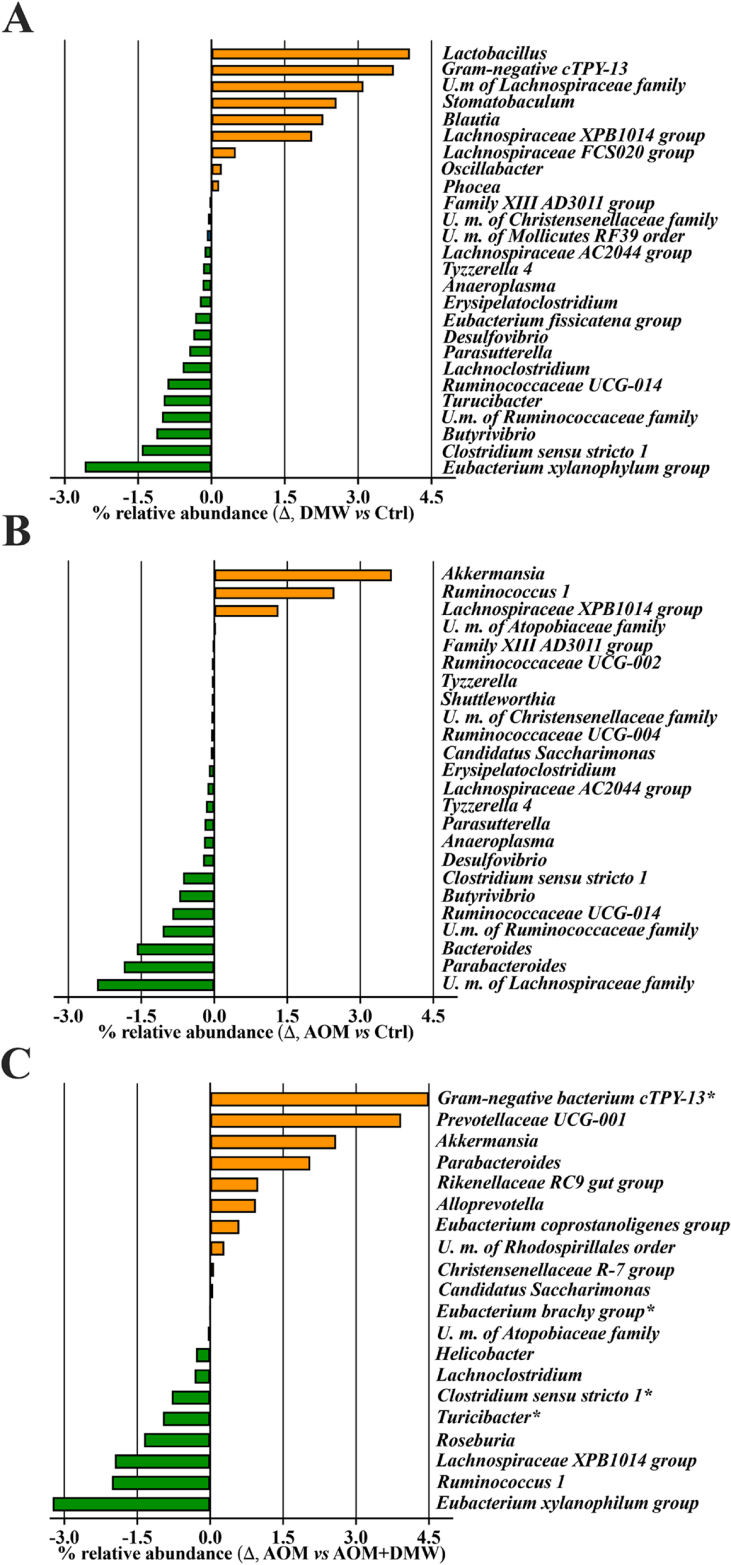
Fecal microbiota composition. Graphs display the genera showing a statistically significant variation (*p* < 0.05) in the delactosed milk whey by-product (DMW) from Mediterranean Italian dairy buffaloes -treated group (**A**) and azoxymethane (AOM)-treated group (**B**) compared with control (untreated mice, at week 13), and in the AOM plus DMW group (**C**) compared with the AOM group (at week 13)

**Fig. 4:**
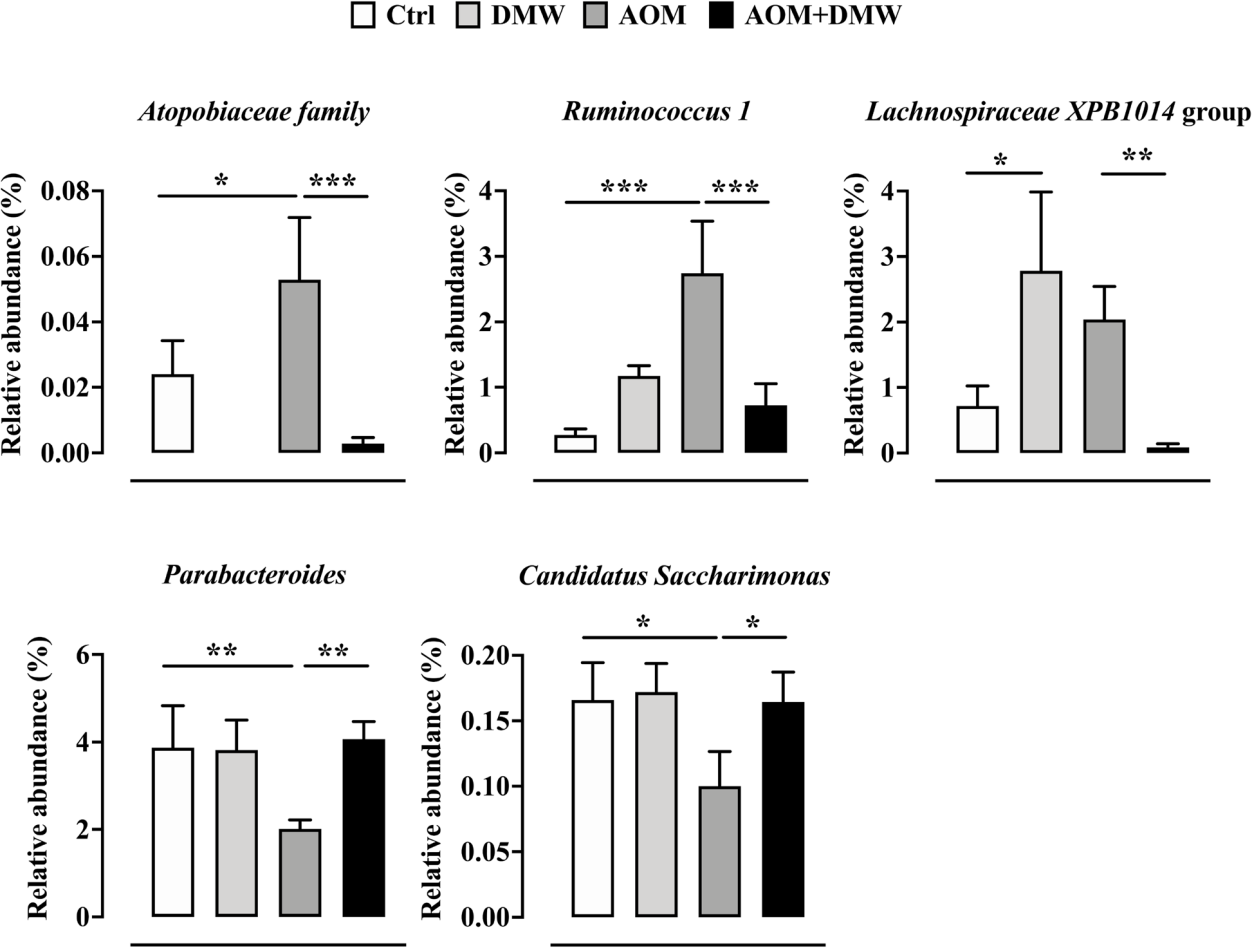
Taxa affected by the azoxymethane (AOM) treatment and restored by delactosed milk whey by-product (DMW) from Mediterranean Italian dairy buffaloes. Graphs reporting the relative abundance of the genera altered by AOM. Data represent the mean ± SEM of 5/7 mice; *p < 0.05, **p < 0.01 and ***p < 0.001

**Fig. 5:**
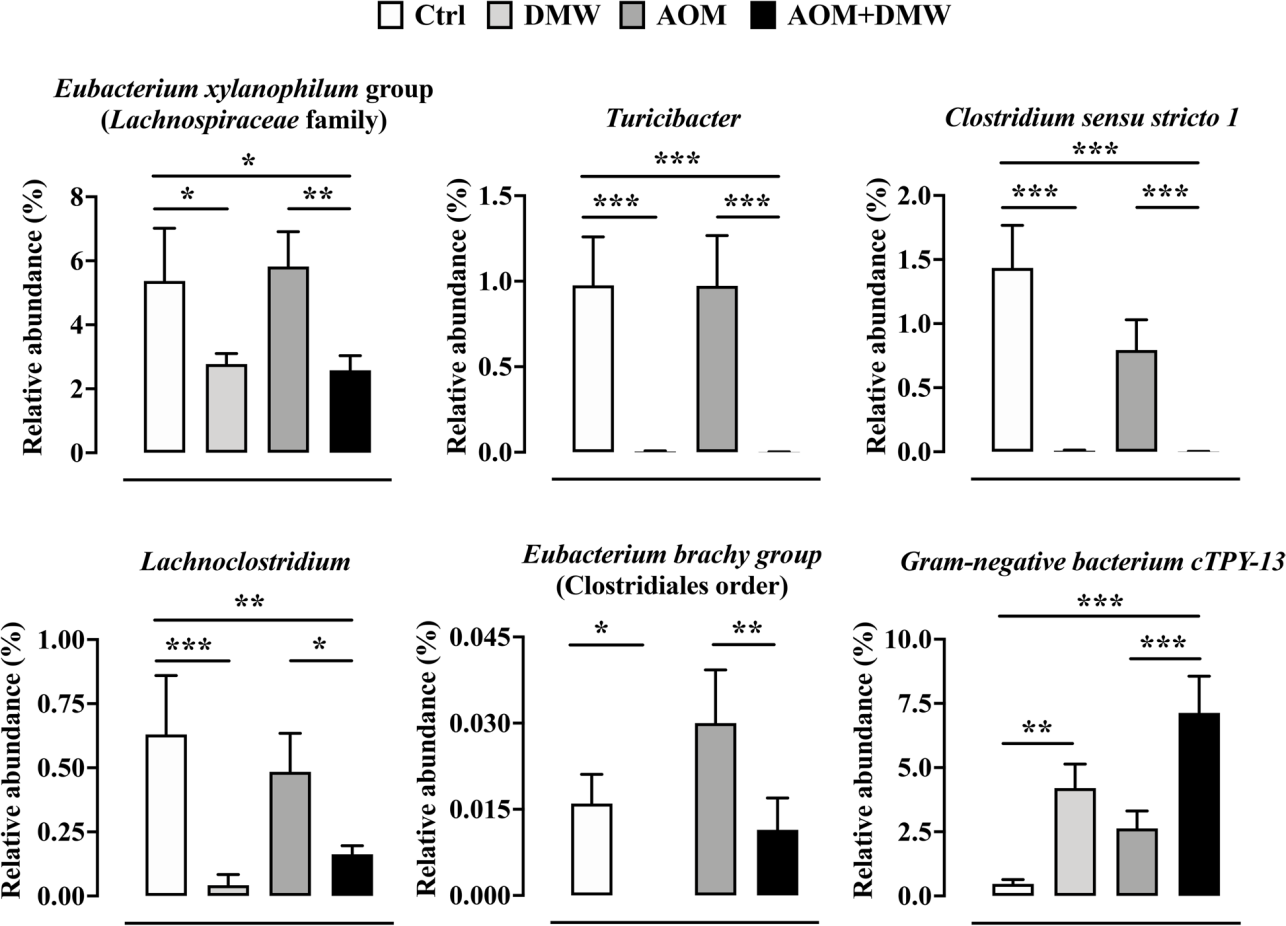
Taxa affected by delactosed milk whey by-product (DMW) from Mediterranean Italian dairy buffaloes. Graphs reporting the relative abundance of the genera altered by DMW. Data represent the mean ± SEM of 5/7 mice; *p < 0.05, **p < 0.01 and ***p < 0.001

**Fig. 6:**
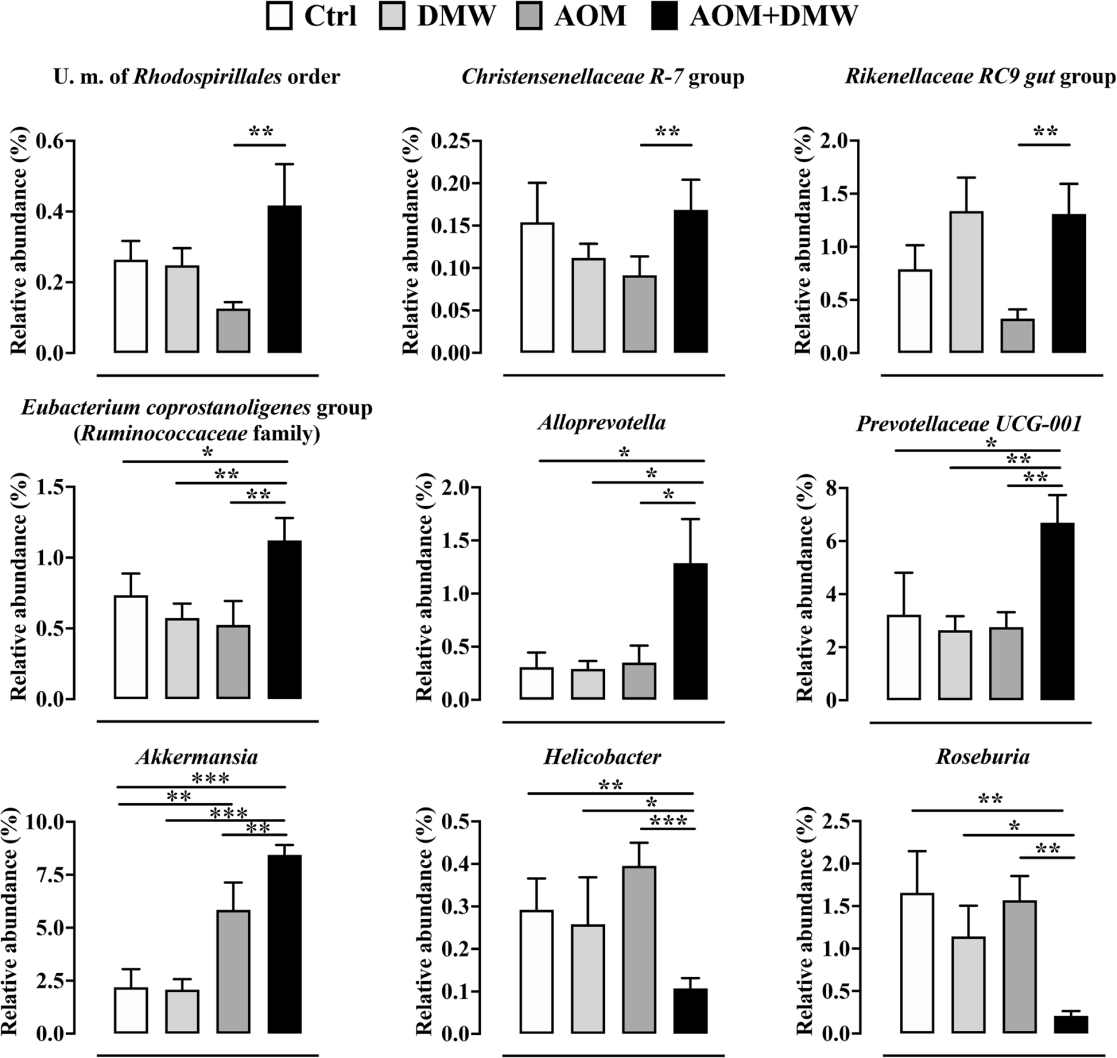
Other taxa differentially represented between azoxymethane (AOM) and AOM plus delactosed milk whey by-product (DMW) from Mediterranean Italian dairy buffaloes. Graphs showing statistically significant differences in various taxa specifically altered by AOM and AOM + DMW groups. Data represent the mean ± SEM of 5/7 mice; *p < 0.05, **p < 0.01, ***p < 0.001

To characterize intestinal microbial composition, 16S-based sequencing analysis was performed on total DNA extracted from mice fecal samples. PCoA analysis based on Bray–Curtis distance revealed that the fecal microbiota in 18 of 24 untreated mice (week 0) clustered together, with 6 mice outside the cluster (indicated by arrows in Supplementary Figure 1). The microbiota composition in the feces of the mice (week 13) for the DMW, AOM and AOM + DMW treatments clustered independently and showed only partial overlap, clearly indicating that the different treatments influenced the fecal microbiota composition (Supplementary Figure 1). The ASVs representation curves showed that the microbial diversity of the samples was completely covered (Supplementary Figure 2 A), while the alpha-diversity analysis revealed similar diversity in the control and treated mice as measured by the Chao1 and Shannon indexes (Supplementary Figure 2 B and C). At week 13, the fecal microbiota composition of untreated mice (control group) did not cluster with that of the same animals at time 0 (week 0 versus week 13, Supplementary Figure 1). This finding suggests a possible influence of age on microbial composition. The analysis of the total microbiota at the phylum, family and genus levels is shown in Supplementary Figure 3. When restricting the analysis to the bacterial genera with relative abundances greater than 0.05%, the genus composition of the fecal microbiota is represented differently in DMW and AOM mice (Fig. 3). Specifically, DMW or AOM treatment (at week 13) alone altered (*p* < 0.05) the microbial composition of 26 (DMW) and 24 (AOM) genera compared with the control group (at week 13) (Fig. 3A and B). Analysis of the genera in the fecal microbiota of the AOM plus DMW group compared to the AOM group also revealed that 20 genera differed (*p* < 0.05) between the two groups (Fig. 3C). Of these 20 genera, five genera were altered by AOM (compared to control) and were restored to control levels by DMW, indicating that DMW counteracted the AOM effect (Fig. 4). Notably, the relative abundance of the three genera *Atopobiaceae, Ruminococcus 1* and *Lachnospiraceae XPB1014*, which was increased in AOM-treated mice, was maintained at the control level by DMW treatment (Fig. 4). An opposite trend was observed for members of the genera *Parabacteroides* and *Candidatus Saccharimonas*, whose relative abundance decreased in AOM-treated mice and was maintained at the control level by DMW treatment (Fig. 4). Another six genera were specifically affected by DMW treatment, and the DMW-induced changes persisted in the presence of AOM (Fig. 5). For five genera, DMW treatment decreased their abundance compared to the control group, and these low abundances were maintained in the AOM plus DMW group (Fig. 5). In the case of the *Eubacterium brachy* group, the DMW-induced reduction (compared to the control) was no longer significant in the AOM plus DMW group (vs control), although an effect of DMW was observed in suppressing the increasing trend of AOM (Fig. 5). The gram-negative bacterium cTPY-13 was increased in DMW treated mice (Fig. 5). For the remaining nine genera that differed between the AOM and AOM plus DMW groups, eight genera were not altered by administration of AOM or DMW alone (compared to control) (Fig. 6). For three out of nine genera, an increase in genus abundance was observed only in the AOM plus DMW group compared with the AOM group (Fig. 6). For the other six genera, differences in genus abundance were observed between the AOM plus DMW group and all other groups (Fig. 6). Of these six genera, four genera were increased by AOM plus DMW (Fig. 6) while the remaining two genera *(Helicobacter* and *Roseburia*) were reduced (Fig. 6). AOM alone caused an increase in the genus *Akkermansia* which was further enhanced by simultaneous treatment with DMW (Fig. 6).

### Effect of AOM and DMW on the blood metabolome

#### Short chain fatty acids (SCFAs)

Analysis of the fecal microbiota had shown that AOM and DMW induced changes in the genera that produce SCFAs. AOM treatment was associated with an increase in butyric and propionic acid, in the blood, with a significant effect for butyric acid (Supplementary Figure 4). DMW administration returned SCFAs to levels comparable to those of the control group (Supplementary Figure 4). No effect of acetic acid has been observed (Supplemenrary Figure 4).

#### Untargeted metabolomic analysis

After extraction for SCFAs quantification, the serum samples were subjected to LC–MS analysis. The resulting total ion chromatograms (TIC) and mass spectra were used for putative identification of compounds by comparing the spectra with data from the literature, freely available databases and /or, where possible, by comparison with authentic standards. The raw data obtained by LC–MS analysis were subjected to statistical analysis (both univariate and multivariate analyses). The statistically different metabolites are listed in Table 3 together with their differential accumulation obtained after comparing the samples treated with AOM and the sample treated with AOM plus DMW. The graphs in Supplementary Figure 5 represent the principal component analysis (PCA) and show a clear separation of the samples depending on the treatment applied. Specifically, in supplementary Figure 5A, the untreated mouse serum (control group) extracted with petroleum ether is separated from the treated groups along component 1. Separation between the treated samples is evident along component 2, with the DMW and AOM plus DMW groups closer together. The PCA result on mice sera extracted with ethyl acetate is reported in Supplementary Figure 5B. In this case, the control group is separated from the treated samples along PC-2, while all treated groups cluster together except for a subgroup spreading along PC-1, due to biological variability. Metabolic pathways altered by DMW treatment included glycerophospholipid metabolism, which is associated with colorectal cancer risk [39, 40]. The predominant alterations observed in AOM + DMW involved the choline-containing phospholipids, namely phosphatidylcholines and lysophosphatidylcholines, with down-regulation of glycerophosphocholine and up-regulation of lysophosphatidylcholine (1-palmitoyl-sn-glycero-3-phosphocholine, 1-octadecenoyl-sn-glycero-3-phosphocholine, 1-eicosapentaenoyl-glycero-3-phosphocholine, and 1-docosahexaenoyl-glycero-3-phosphocholine).

**Table 3:** Putatively identified metabolites that are differentially accumulated in sera from mice treated with azoxymethane (AOM) and delactosed milk whey by-product (DMW) *vs* AOM

Compound	RT (min)	Monoisotopic Experimental Mass (Da)	Monoisotopic Theoretical Mass (Da)	Molecular formula	AOM + DMW *vs* AOM
5-Methylcytidine	1.175	257.1019	257.10117	C_10_H_15_N_3_O_5_	down
Glycerophosphocholine	1.182	257.1029	257.10282	C_8_H_20_NO_6_P	down
1-Palmitoyl-sn-glycero-3-phosphocholine LysoPC(16:0/0:0)	6.510	495.3339	495.33248	C_24_H_50_NO_7_P	up
1-(9Z,12Z-octadecadienoyl)-sn-glycero-3-phosphocholine LysoPC(18:2(9Z,12Z)/0:0)	10.222	519.3333	519.33248	C_26_H_50_NO_7_P	down
1-[(11Z)-Octadecenoyl]-sn-glycero-3-phosphocholine LysoPC(18:1(11Z)/0:0)	6.888	521.3495	521.34814	C_26_H_52_NO_7_P	up
1-octadecanoyl-sn-glycero-3-phosphocholine LysoPC(18:0/0:0)	7.573	523.3634	523.36379	C_26_H_54_NO_7_P	down
1-Eicosapentaenoyl-glycero-3-phosphocholine LysoPC(20:5(5Z,8Z,11Z,14Z,17Z)/0:0)	8.146	541.3166	541.31683	C_28_H_48_NO_7_P	up
1-Eicsoatetraenoyl-glycero-3-phosphocholine LysoPC(20:4(8Z,11Z,14Z,17Z)/0:0)	9.499	543.3334	543.33248	C_28_H_50_NO_7_P	down
1-Docosahexaenoyl-glycero-3-phosphocholine LysoPC(22:6(4Z,7Z,10Z,13Z,16Z,19Z)/0:0)	9.081	567.3337	567.33248	C_30_H_50_NO_7_P	up

*Down* Down regulated, *Up* Up regulated

### In vitro cytotoxicity

#### Effect of DMW/WF on cell viability

**Fig. 7:**
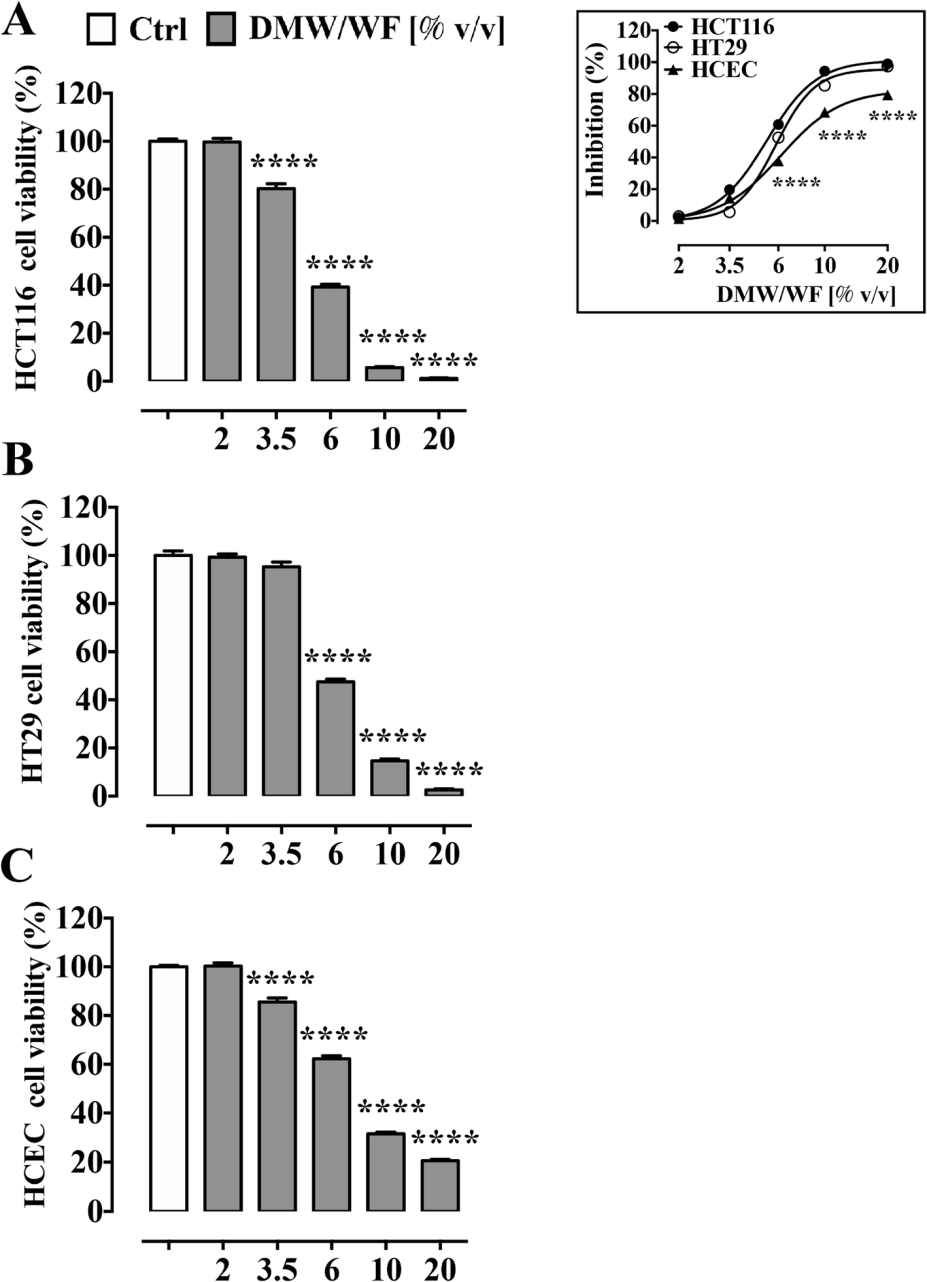
Delactosed milk whey by-product without fat (DMW/WF) from Mediterranean Italian dairy buffaloes reduces cell viability in human colorectal cancer cell lines HCT116 and HT29. HCT116 (**A**) and HT29 (**B**) and a healthy human colonic epithelial cell line HCEC (**C**) were cultured in the absence or presence of increasing concentrations of DMW/WF (2–20% v/v) for 24 h. The viability rate of DMW/WF-treated cells was measured using the MTT assay, as described in Material and Methods. The insert shows the difference between the curves representing the inhibitory effect of DMW/WF in HCT116, HT29 and HCEC. Data are expressed as mean ± SEM of three independent experiments. ****p < 0.0001 *vs* control (untreated cells)

The viability of HCT116 cells was inhibited by DMW/WF (2–20% v/v) in a concentration-dependent manner (Fig. 7A). After 24 h-treatment with DMW/WF, the 3.5% v/v, 6% v/v, 10% v/v and 20% v/v concentrations reduced HCT116 cell viability by 19.7%, 60.7%, 94.4%, and 98.9%, respectively. Similar results were obtained using HT29 cells (Fig. 7B). DMW/WF-treated non-tumor HCEC cells were less sensitive as cell viability decreased by 14.1%, 37.8%, 68.5% and 79.5% after 3.5% v/v, 6% v/v, 10% v/v and 20% v/v DMW/WF concentrations, respectively (Fig. 7C). The EC_50_ was 5.27 ± 1.02 v/v, 5.84 ± 1.01 v/v and 6.17 ± 1.03 v/v for HCT116, HT29 and HCEC cells, respectively. The E_max_ was 101.1 ± 1.7%, 95.85 ± 1.29%, and 82.3 ± 1.03% for HCT116, HT29 and HCEC cells, respectively.

#### Effect of DMW/WF on HCT116 cell morphology

**Fig. 8:**
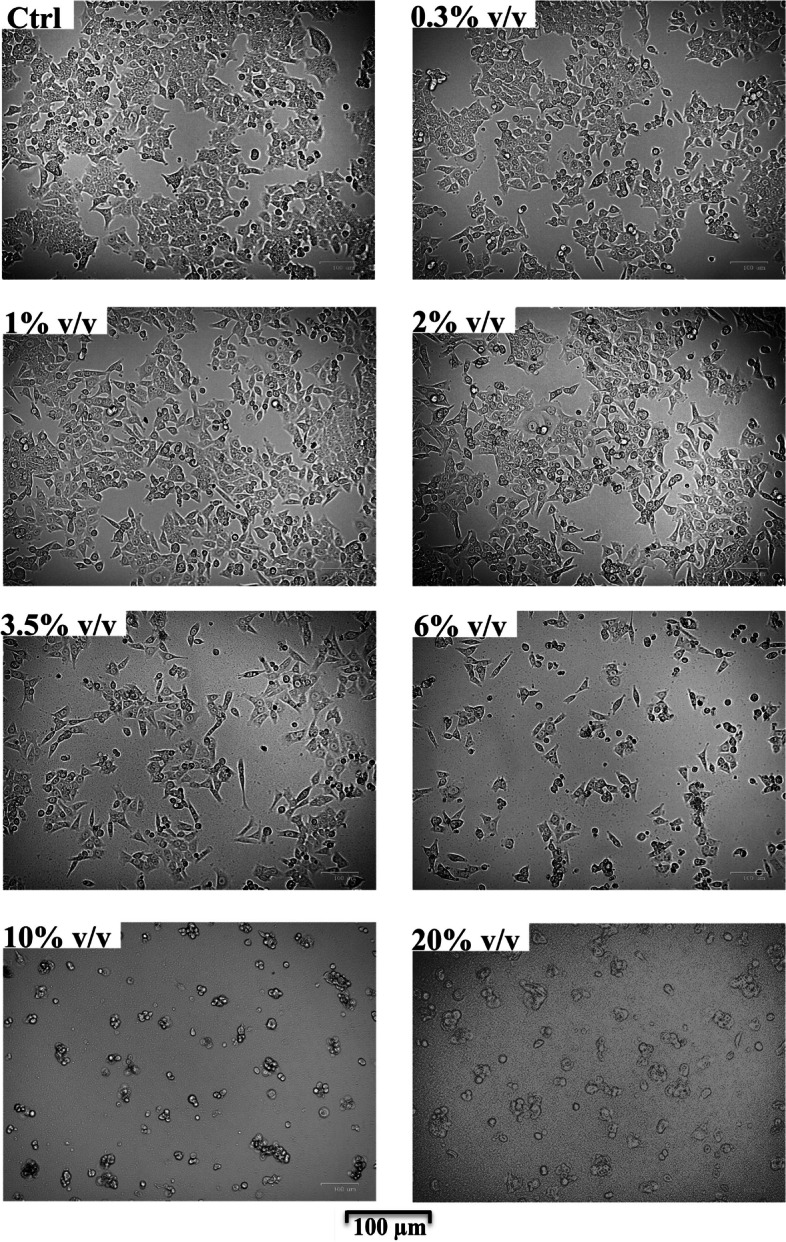
Delactosed milk whey by-product without fat (DMW/WF) from Mediterranean Italian dairy buffaloes triggers phenotypic changes in the human colorectal cancer cell line HCT116. Representative bright field images indicating morphological changes in HCT116 cells after 24 h incubation with or without increasing concentrations of DMW/WF (0.3–20% v/v). Cell morphology was evaluated by fluorescence microscopy using a Zoe™ Fluorescent Cell Imager (Biorad). Magnification, × 100

Untreated HCT116 cells (control) or cells exposed to 0.3% v/v DMW/WF spread regularly in the cell culture multiwell plates and grew to semiconfluence (Fig. 8). In contrast, HCT116 cells treated with 1–2% v/v DMW/WF exhibited a thin and flattened shape compared to control cells (Fig. 8). Treatment with 4% v/v or 6% v/v DMW/WF was associated with a decrease in cell size; cells shrank and lost contact with neighboring cells (Fig. 8). Cells treated with the highest DMW/WF concentrations (10% or 20% v/v) were very small and rounded (hallmarks of apoptotic cells), detached from the surface of the culture plate, and floated in the cell culture medium (Fig. 8).

### Effect of DMW/WF on HCT116 cell apoptosis

**Fig. 9:**
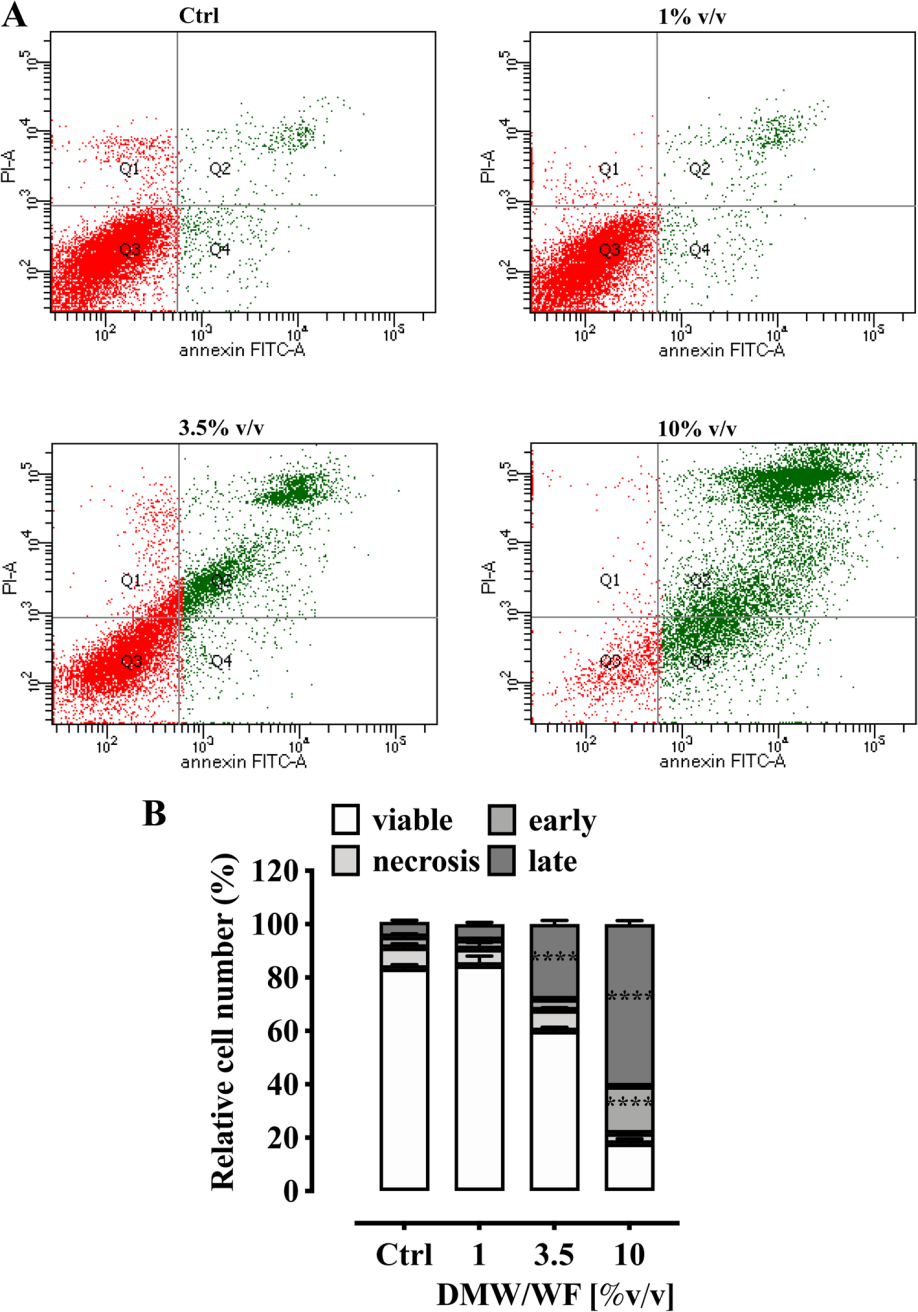
Delactosed milk whey by-product without fat (DMW/WF) from Mediterranean Italian dairy buffaloes induces apoptosis in the human colorectal cell line HCT116. Flow cytometric analysis of apoptosis in HCT116 cells untreated or treated with DMW/WF (1–10% v/v concentrations) for 24 h. **A** Representative dot plot showing Annexin-V-FITC-/PI-(lower left quadrant/viable cells), annexin-V-FITC + /PI − (lower right quadrant/early apoptotic cells), Annexin-V-FITC + /PI + (upper right quadrant/late apoptotic cells) and Annexin-VFITC -/PI + (upper left quadrant/necrotic cells) in HCT116 cells as acquired by flow cytometry. **B** percentage of viable, apoptotic, and necrotic cell populations in HCT116 cells after 24 h incubation period with medium alone (untreated) or with DMW/WF (1–10% v/v). Data are presented as mean ± SEM of three independent experiments. ****p < 0.0001 vs control (Ctrl, untreated cells)

To determine whether the cytotoxic effect of DMW/WF was due to apoptotic or necrotic effects, we performed an Annexin V/PI double staining assay on HCT116 cells treated with different DMW/WF concentrations (0–10% v/v) for 24 h. Exposure of HCT116 cells to 1% v/v DMW/WF resulted in no change in the apoptotic population at both early (Annexin V + /PI −) and late (Annexin V + /PI +) stages (Fig. 9). In contrast, cells treated with 4% v/v or 10% v/v DMW/WF showed a significant increase in apoptotic/necrotic population at early and/or late stages (fold increase: DMW/WF 4% v/v 28.3 ± 1.3%* for apoptotic population at late stage; DMW/WF 10% v/v 17.7 ± 0.6%* and 60.7 ± 1.3%* for apoptotic population at early and late stages, respectively, *p < 0.0001 *versus* untreated cells) (Fig. 9).

To determine whether DMW/WF impaired the viability of healthy cells (i.e., HCEC) inducing apoptosis, we performed flow cytometric analysis (Supplementary Figure 6). We demonstrated that treatment of HCEC cells with 1% v/v or 3.5 v/v DMW/WF did not increase early and late apoptotic cell populations (Supplementary Figure 6A). In contrast, exposure of HCEC cells to 10% v/v DMW/WF resulted in a decrease in the percentage of viable cells (77.3% ± 1.445 DMW/WF versus 94.6 ± 1.905% ctrl, ****p < 0.0001) and a concomitant increase in both early (5.8% ± 0.360 DMW/WF versus 1.18% ± 0.410 ctrl, **p < 0.01) and late (15.16 ± 0.525% DMW versus 2.675 ± 0.415% ctrl, ****p < 0.0001) apoptotic cell populations (Supplementary Figure 6A, B). Compared with HCEC cells, HCT116 cells showed 3.7- and 5.3-fold higher sensitivity to DMW/WF treatment at concentrations of 3.5% v/v and 10% v/v, respectively, confirming a safe profile of DMW/WF (Supplementary Figure 6C).

**Fig. 10:**
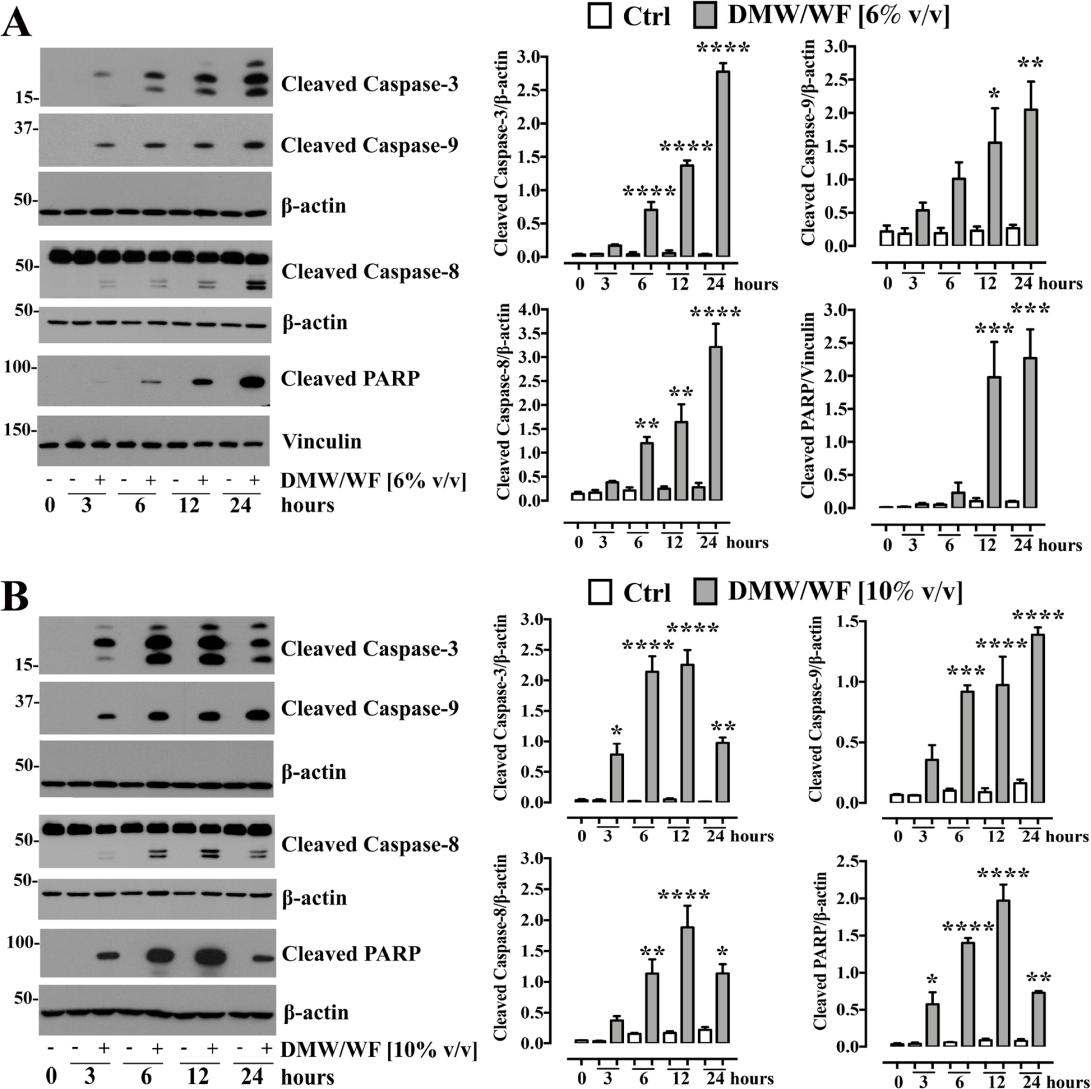
Delactosed milk whey by-product without fat (DMW/WF) from Mediterranean Italian dairy buffaloes activates apoptosis-related protein expression in the human colorectal cell line HCT116. Representative images of the protein expressions of cleaved caspase-3, caspase-9, caspase-8 and poly (ADP) ribose polymerase (PARP) measured by western blot analysis. HCT116 cells were treated or not with DMW/WF [6% v/v (**A**) and 10% v/v (**B**) at different time points (0–24 h)]. The right side of the panel shows the results of immunoblot densitometric analysis of the protein expression levels in HCT116 cells (at different time points) after DMW/WF (6% v/v or 10% v/v concentrations) treatment. Cleaved caspase-3, caspase-9, caspase-8 and PARP signals were normalized on the housekeeping proteins β-actin or vinculin. Data are expressed as mean ± S.E.M of three independent experiments. *p < 0.05, **p < 0.01, ***p < 0.001, and ****p < 0.0001 *vs* control (Ctrl, untreated cells)

To better understand the mechanism by which DMW/WF-treated HCT116 cells undergo apoptosis, we performed a time course (0–24 h) and concentration-dependent experiment (6% v/v or 10% v/v DMW/WF concentrations). Exposure of HCT116 cells to 6% v/v DMW/WF resulted in a time-dependent increase (*p* < 0.05–0.0001) in caspase-3, caspase-9, caspase-8, and PARP cleaved fragments (Fig. 10). At the highest concentration (10% v/v), the increase in the caspases- and PARP- cleaved fragments was more pronounced but not time-dependent, suggesting that the time at which caspases and PARP cleavages occurred was related to both concentration and exposure time (Fig. 10 A and B).

## Discussion

We had previously shown that non-protein metabolites in buffalo milk whey by-product have antiproliferative and pro-apoptotic effects on human cancer cell lines in vitro, including colorectal cancer cells [16]. We had also shown in a preliminary study that buffalo milk whey upregulated apoptotic pathways in a HCT116 xenograft mouse model of colorectal cancer [19]. We have now extended our previous work by examining the effects of delactosed buffalo milk whey by-product (DMW) in an in vivo model of CRC. We provided evidence that DMW treatment exerts chemopreventive effects by reducing tumor formation and this effect was associated to changes in the gut microbiota and blood markers. Moreover, we demonstrated that defatted DMW exerted a strong cytotoxic effect on human colorectal cancer cell lines by affecting both the extrinsic and intrinsic apoptotic pathways thus providing compelling evidence of a possible valuable role of DMW in colorectal carcinogenesis development.

The development of CRC occurs in a multistage process in which healthy epithelium slowly evolves into preneoplastic lesions, namely aberrant crypt foci (ACF), which over time develop into polyps and eventually malignant carcinomas. In this study, using the AOM mouse model of CRC, which induces the formation of CRC similar to the pathogenesis of sporadic human CRC, we showed that orally administered DMW significantly reduced the percentage of mice with ACF containing more than four crypts. These are the early precancerous lesions that more often progress to CRC. In addition, DMW also reduced the percentage of mice with tumours, mice with more than one tumour and tumour growth induced by AOM. AOM treatment alone was associated with increased mitotic figures in colon tissue, which were reduced in mice receiving pre-treatment with DMW. These results provided compelling evidence that buffalo DMW reduces the incidence and severity of colorectal cancer.

Mechanistically, we found increased expression of cleaved caspase-3, caspase-9 and PARP-1 in the colon tissue of AOM-treated mice, which was significantly reduced by DMW. It is generally believed that increased expression of caspase-3 correlates with the induction of apoptosis and therefore caspase-3 is considered an indicator of cancer treatment efficacy [41]. However, there is increasing evidence for a non-apoptotic role of caspase-3 in cancer-induced cell migration, invasiveness, tumor angiogenesis and stress-induced growth of cancer cells [42–45]. In addition, it has been reported that colorectal cancer patients with high expression of caspase-3 have a worse prognosis than patients with lower expression [46–49]. In view of these findings, our results suggest a pro-tumor role of caspase-3 in the AOM-induced colon cancer model. Few data are available for the potential role of caspase-9 in malignancies, although increased expression of caspase-9 has been reported in lung cancer progressing to a metastatic phenotype [50] and in colorectal cancer patients with a poor prognosis [51]. PARP-1 has been shown to play a role in the development of colorectal cancer, as PARP-1 expression is significantly higher in colorectal cancer than in healthy tissue and directly correlates with tumor size and histopathology [52, 53]. In a colitis-associated colorectal cancer model, AOM was associated with greater tumor number in wild-type mice than in PARP-1^−/−^ mice [54]. Our results on PARP-1 expression (Western blot and immunofluorescence) are consistent with these data, confirming its tumor-promoting "burden".

The gut microbiota has been shown to be a critical factor in the development, progression and metastasis of CRC [55–57]. Dysbiosis of the gut microbiota has been found in colorectal cancer patients as well as in mice with spontaneous and chemically induced colon tumorigenesis [58–60]. Here, we found that DMW administration in healthy mice resulted in an increase in bacterial genera known for their beneficial effects. In particular, DMW treatment increased the relative abundance of members of the genera *Eubacterium*, *Lactobacillus*, *Prevotellaceae* and *Stomatobaculum*, which are well-established probiotics with health-promoting roles and strictly anaerobic species capable of producing short-chain fatty acids (SCFA) such as butyrate and propionate, respectively [61–63]. In addition, DMW supplementation also led to an increase in *Blautia* members, which were recently identified as a new genus with 20 species. Remarkably, bioinformatic analysis of 12 *Blautia* species has enabled the identification of potential probiotics capable of preventing infections, improving glucose and lipid homeostasis and reducing obesity-related disorders [64–66]. Similarly, an increase in the gram-negative bacterium *cTPY-13*, which belongs to the *Bacteroides* class and was previously identified in the large intestine of mice, was observed in mice treated with DMW [67]. Finally, we also found that DMW caused a decrease in members of the genera *Turicibacter*, *Eubacterium*, *Clostridium* and *Lachnoclostridium*, whose presence in the gut was previously associated with inflammation and cancer development [68]. Remarkably, the DMW-induced changes in the gut microbiota were maintained after treatment with AOM. Taken together, all these data suggest that DMW supplementation has a positive effect on the gut microbial community, leading to an enrichment or depletion of genera associated with health or disease status.

According to the literature [69], administration of the carcinogenic agent AOM resulted in dysbiosis of the gut microbiota, which was partially restored by DMW in mice. Specifically, AOM administration led to an increase in three gram-positive genera, namely *Atopobiaceae*, *Ruminococcus 1* and *Lachnospiraceae XPB1014*, and this effect was reversed by DMW. Remarkably, elevated levels of these three genera were found in CRC and/or in patients with squamous cell carcinoma of the oesophagus, but not in healthy individuals [63, 70], suggesting a detrimental role of these bacteria in the development of CRC. An opposite trend was observed for members of the genera *Parabacteroides* and *Candidatus Saccharimonas*, whose relative abundance decreased in AOM-treated mice and was completely reversed by DMW treatment. The role of *Parabacteroides*, gram-negative bacteria commonly found in the gut of healthy animals, is controversial as their abundance has been associated with health and disease status. Some reports have found high abundance of *Parabacteroides* in CRC patients [70, 71], while other papers have reported a negative correlation between *Parabacteroides* and CRC development [72, 73]. Members of the genus *Candidatus Saccharimonas* have been shown to be greatly reduced in inflammatory diseases [74] and to suppress the production of inflammatory markers in macrophages, indicating an immunosuppressive effect [75].

The taxa that are increased in the faeces of AOM plus DMW-treated mice are genera known for their health-promoting effects in the gut. For example, *Eubacterium spp*. can produce SCFA and thus contribute to the modulation of intestinal inflammation [76]. In particular, the species *E. coprostanoligenes* has recently received much attention for its protective effects against colitis and carcinogenesis [76]. In addition, members of the *Prevotellaceae* taxa play an important role in the degradation of complex dietary polysaccharides, leading to the production of SCFA, the increase of which has been reported after the use of probiotics [62, 63]. In agreement with a recent article [77], our results showed that administration of AOM led to an increase in the genus *Akkermansia*. In the present study, this increase was further enhanced by AOM + DMW. *Akkermansia* is considered an important symbiont of the gut microbial population, and its abundance is associated with health-promoting effects. Interestingly, high Akkermansia levels were found in CRC patients compared to healthy individuals and increased after chemotherapy, suggesting a positive correlation with therapeutic effect [78, 79]. Two genera, *Helicobacter* and *Roseburia*, were specifically reduced by DMW in AOM-treated mice. Species of the genus *Helicobacter*, which are able to colonise the intestinal environment, are commonly associated with diarrhoea and the development of inflammation and CRC [80]. Therefore, the DMW-induced *Helicobacter* reduction in AOM-treated mice supports our hypothesis that the antitumour effect of DMW is partly due to the shift in the composition of the gut microbiota towards a "healthier" community. Strikingly, however, our results also showed a decrease in the abundance of *Roseburia* (considered a health-promoting genus that can produce butyrate and other SCFAs [81]) in mice with tumours treated with DMW. Since a decrease in *Roseburia* bacteria has been reported in response to increased carbohydrate intake [82, 83], we can speculate that the presence of carbohydrates in DMW may be responsible for the effect of DMW on *Roseburia* abundance. The above findings highlight that the gut microbiota is influenced by many factors and explain why it can be difficult to establish clear links between a particular treatment, the microbiota and colorectal cancer [84].

Considering the effect of DMW on bacteria producing SCFAs, in this study we also measured the content of SCFAs in mouse sera. SCFAs, which include acetate, butyrate and propionate, are the major microbial end products of the fermentation process in the colon. Of these, butyrate has been reported to have a beneficial effect against cancers, including colorectal cancer [85, 86]. However, it has also been reported that the effect of butyrate varies depending on the dosage. Low doses of butyric acid stimulated the proliferation of colon epithelial cells, while it was inhibited at high concentrations [87]. In our study, we show increased butyric acid levels in the sera of AOM-treated mice, which were restored to baseline levels by DMW, probably due to the effect of DMW on the incidence of *Roseburia*. The effect of DMW on butyric acid levels seems to indicate a beneficial effect of butyric acid on the development of colon cancer. Further experiments are needed to clarify the role of butyric acid in the development of CRC triggered by AOM.

Further evidence for the preventive effect of DMW on the development of CRC came from the untargeted metabolomic analysis performed on mouse sera. AOM treatment resulted in an increase in two lipophospholipids known to be potential colorectal cancer biomarkers ([88–90]. DMW was able to lower the lipophospholipid levels increased by AOM.

We also investigated the cytotoxic effect of DMW without fat (DMW/WF) on isolated CRC cell lines as well as on a healthy human colonic epithelial cell line (HCEC). We demonstrated that DMW/WF treatment affected the viability of both CRC cell lines (HCT116 and HT29) and, to a lesser extent, HCEC in a concentration-dependent manner, suggesting a more selective effect on CRC cells than on healthy colonic epithelial cells. Since δ-VB has been reported to exert a cytotoxic effect on HT-29 cells, it could be hypothesized that the cytotoxic effect of DMW is due to the presence of this compound. However, such a hypothesis can be ruled out as our results showed that 20% v/v DMW (containing 55.5 µM δ-VB) inhibited cell viability by ~ 99%, which is not in agreement with our previous results where δ-VB inhibited cell viability by ~ 30% [17]. As further confirmation that the cytotoxic effect of DMW is not only due to δ- VB (but also to other compounds), a recent study showed that a purified fraction (3KDa) of whey by-product containing δ-VB (138 mM) exerted a less pronounced cytotoxic effect than our results (cell viability by ~ 30%) [16].

Morphological analysis of the cells showed that the DMW/WF-induced cytotoxic effect is probably due to an apoptotic cell death process, as the phenotypic changes (i.e. cell shrinkage, fragmentation into membrane-bound apoptotic bodies) are features of an apoptotic process. These data were further supported by the flow cytometry assay, which showed that treatment of HCT116 cells with DMW resulted in an increase in early and/or late apoptotic events without significant necrotic signs.

To determine the molecular pathway involved in DMW/WF-induced apoptotic effects in HCT116 cells, we examined the expression of proteins related to apoptosis by Western blot analysis. Apoptosis is a finely tuned process initiated by a cellular intrinsic or/and cellular extrinsic pathway.

Cell death signals from the extrinsic pathway lead to activation of caspase-8, which in turn can directly cleave and activate effector caspases, such as caspase-3. Similarly, the intrinsic apoptosis pathway leads to activation of effector caspases mediated by caspase-9 [91]. Since our results show increased cleavage of caspase-3, -8 and -9 after DMW/WF treatment, the intrinsic and extrinsic pathways may likely be involved in the apoptotic effect of DMW/WF. It is well known that cleaved caspase-3 activates the cleavage of PARP, a well-known cellular hallmark of apoptosis. This fits with our results showing an increase in PARP cleavage.

## Conclusions

In conclusion, the present study provides the first evidence that DMW, a by-product of the dairy processing industry, has chemopreventive effects in an AOM-induced sporadic mouse model of colorectal cancer by reducing the number of tumors, and that this effect is associated with a modulation of the composition of the gut microbiota and blood metabolome that can be considered beneficial to health. Furthermore, we have shown that defatted DMW has a direct cytotoxic effect on human colorectal adenocarcinoma cell lines by triggering a pro-apoptotic mechanism. Identification of the bioactive compounds, which may be a single compound or a mixture of these compounds, is necessary to better understand the mechanisms underlying the chemopreventive effects of DMW and to identify potential candidates for therapeutic strategies targeting the microbiota. Buffalo milk whey by-product has great translational potential as an important dietary supplement (prebiotic) to support a healthy gut microbiota and reduce the prevalence of CRC in humans.

### Supplementary Information

**Additional file 1: Supplementary Figure 1.** Principal Coordinate Analysis (PCoA). Plots generated using weighted UniFrac distance matrix. The different groups are indicated by various colours as indicated. Coloured circles were used to identify the mice before the indicated treatment (at week 0, black circle) and after 13 weeks of treatments with azoxymethane (AOM, yellow circle), Delactosed milk whey by-product (DMW, green circle) and AOM plus DMW (AOM+DMW, pink circle). Arrows identify 6 individuals outside of the clusters.

**Additional file 2: Supplementary Figure 2.** Alpha diversity rarefaction plots. Estimation of the microbial taxa richness and diversity in fecal samples, based on Chao 1 A) and Shannon B) indexes. C) The number of observed ASVs in each sample is reported.

**Additional file 3: Supplementary Figure 3.** Fecal microbiota composition. Bar plots reporting the relative Amplicon Sequence Variant (ASV) abundance at the A) phylum, B) family, and C) genus levels, as mean values within each group. Only Taxa represented by ASVs abundance >1% have been considered for the analysis.

**Additional file 4: Supplementary Figure S4.** Oral administration of delactosed milk whey by-product (DMW) restores the levels of SCFA affected by azoxymethane (AOM) in serum samples of mice. AOM (40 mg/kg in total, intraperitoneally) was administered, at the single dose of 10 mg/kg, at the beginning of the first, second, third and fourth week. DMW was given (by oral gavage), at the 10 ml/kg dose, three times a week for the whole duration of the experiment starting 1 week before the first administration of AOM. Blood samples were collected by cardiac puncture at the end of experiment (i.e., 13 weeks after the first injection of AOM). Data represent the mean ± SEM of six individual mice sera. #*p*<0.01 *vs* control (Ctrl, untreated mice), *p<0.05 *vs* AOM alone.

**Additional file 5: Supplementary Figure 5.** Principal components analysis (PCA) score plots of LC-MS Q-TOF data of water residues of serum after extraction with petroleum ether (A) and ethyl acetate (B). Each group of replicates subjected to different treatments is depicted with a different color: control group (C) = brown; AOM = red; S = grey; AOM added with S = blu.

**Additional file 6: Supplementary Figure 6.** Delactosed milk whey by-product without fat (DMW/WF) causes weak apoptotic effects in HCEC cells. (A-B) HCEC cells were treated with or without increasing concentrations of DMW/WF for 24 hours. Detached and adherent cells were collected and stained with Annexin V and propidium iodide, and then events for early and late apoptotic cells were counted using the BriCyte E6 system (Mindray, PR China) as described in Material and Methods. Data represent the mean ± SEM of two independent experiments. Statistical analysis was performed using ANOVA followed by Dunnett Post-hoc test to determine statistical significance (**p < 0.01; ****p<0.0001 vs ctrl cells). (C) The graph shows the different behavior of HCT116 and HCEC cells in terms of apoptosis induction after exposure to different concentrations of DMW/WF. ****p<0.0001.

## Data Availability

All data supporting the findings of this study are available within the paper and its Supplementary Information. The 16S rRNA gene sequencing dataset analyzed in this study is publicly available from the ENA database under the accession number PRJEB61962.

## Abbreviations

CRC: Colorectal cancer
DMW: Delactosed milk whey by-product
DMW/WF: Delactosed milk whey by-product without fat
HPLC: High Performance Liquid Chromatograph
PARP: Poly ADP-ribose polymerase
