# Supplementary figures and images for: Chemopreventive effect of a milk whey by-product derived from Buffalo (Bubalus bubalis) in protecting from colorectal carcinogenesis
Source: Cell Commun Signal. 2023 Sep 20;21:245. doi: 10.1186/s12964-023-01271-5 (PMC10510155; doi:10.1186/s12964-023-01271-5)

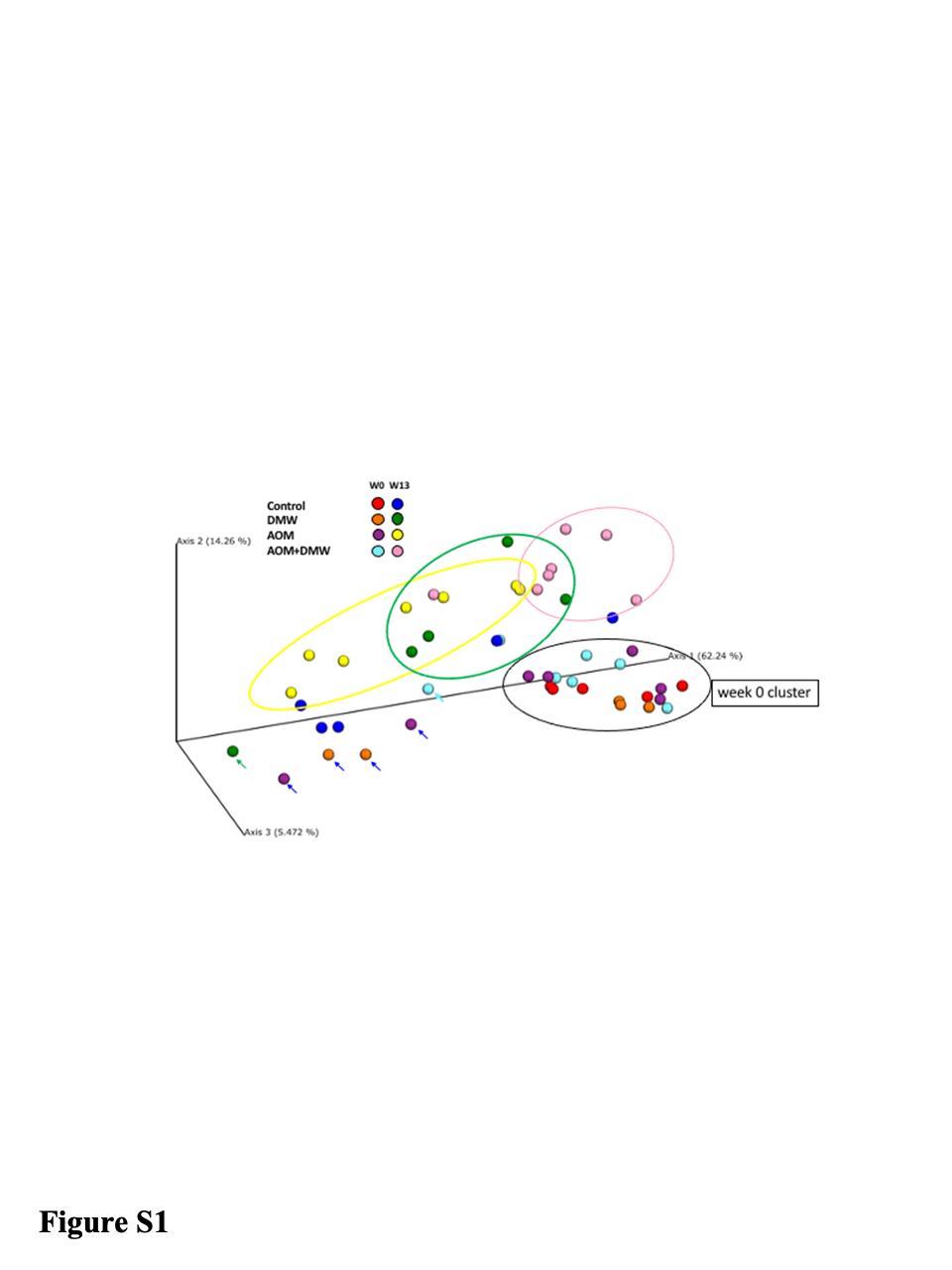

Supplement: Supplementary file 2 — Additional file 1: Supplementary Figure 1. Principal Coordinate Analysis (PCoA). Plots generated using weighted UniFrac distance matrix. The different groups are indicated by various colours as indicated. Coloured circles were used to identify the mice before the indicated treatment (at week 0, black circle) and after 13 weeks of treatments with azoxymethane (AOM, yellow circle), Delactosed milk whey by-product (DMW, green circle) and AOM plus DMW (AOM+DMW, pink circle). Arrows identify 6 individuals outside of the clusters. [file 12964_2023_1271_MOESM1_ESM.tiff]

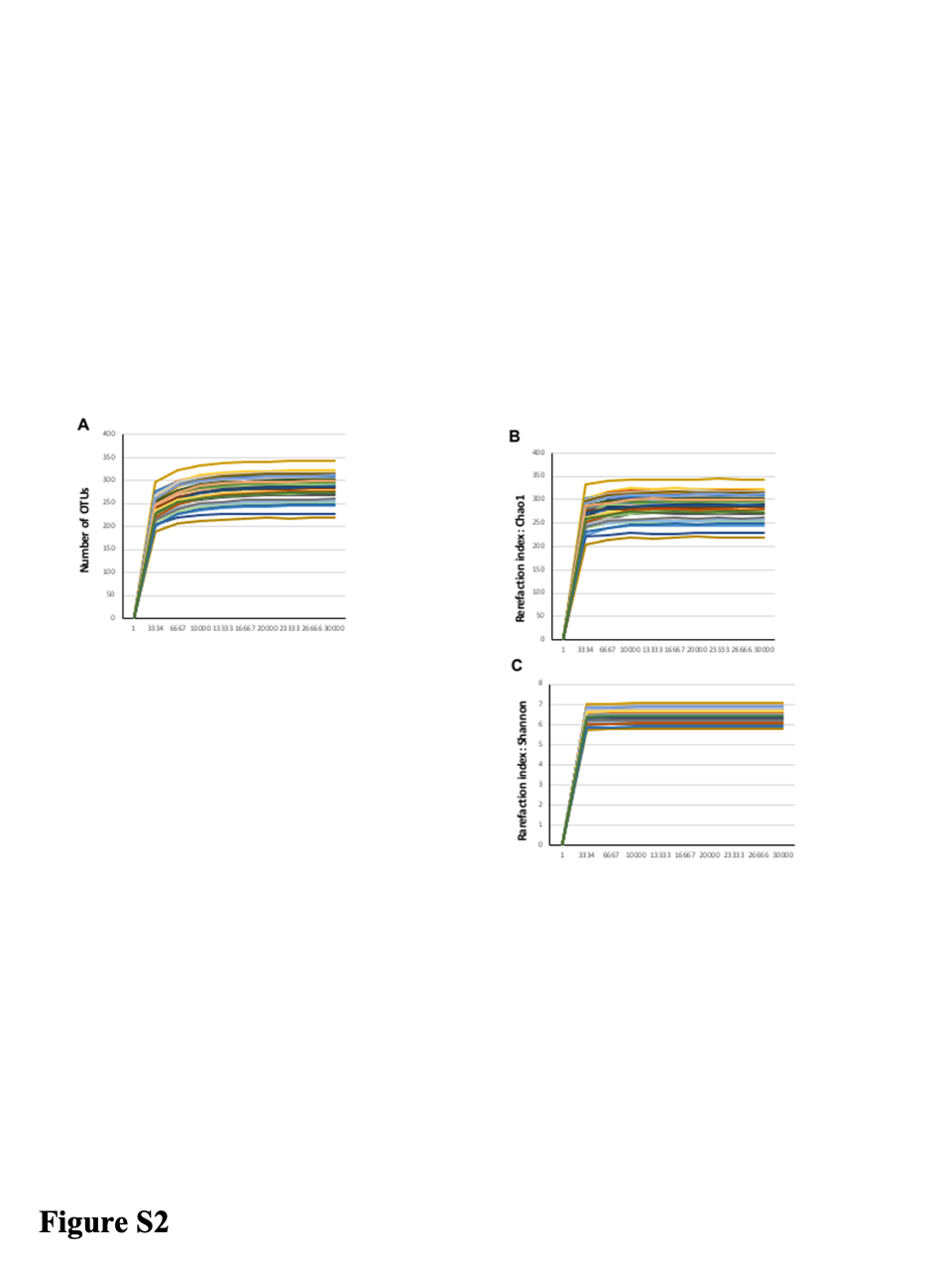

Supplement: Supplementary file 3 — Additional file 2: Supplementary Figure 2. Alpha diversity rarefaction plots. Estimation of the microbial taxa richness and diversity in fecal samples, based on Chao 1 A) and Shannon B) indexes. C) The number of observed ASVs in each sample is reported. [file 12964_2023_1271_MOESM2_ESM.tiff]

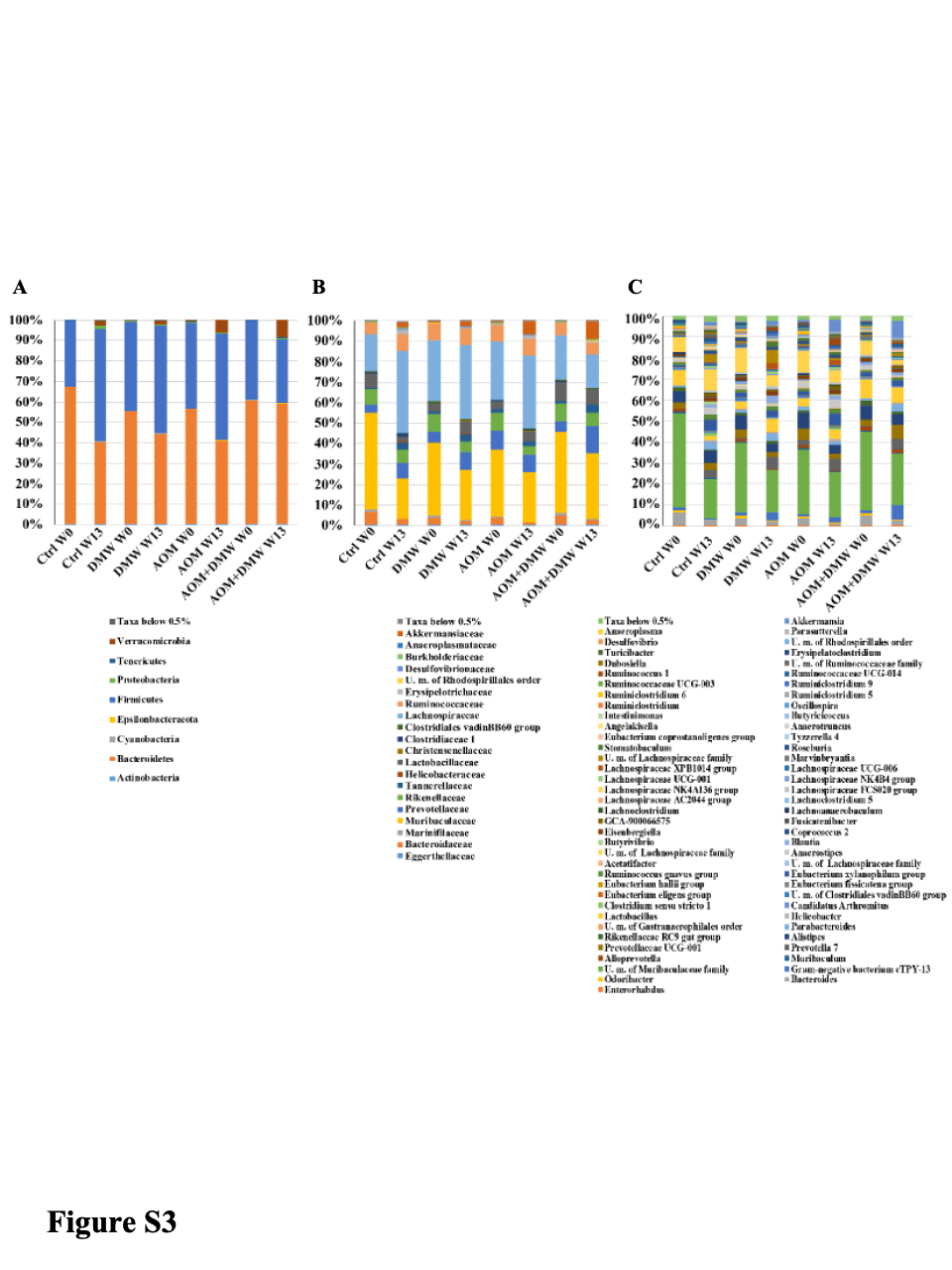

Supplement: Supplementary file 4 — Additional file 3: Supplementary Figure 3. Fecal microbiota composition. Bar plots reporting the relative Amplicon Sequence Variant (ASV) abundance at the A) phylum, B) family, and C) genus levels, as mean values within each group. Only Taxa represented by ASVs abundance >1% have been considered for the analysis. [file 12964_2023_1271_MOESM3_ESM.tiff]

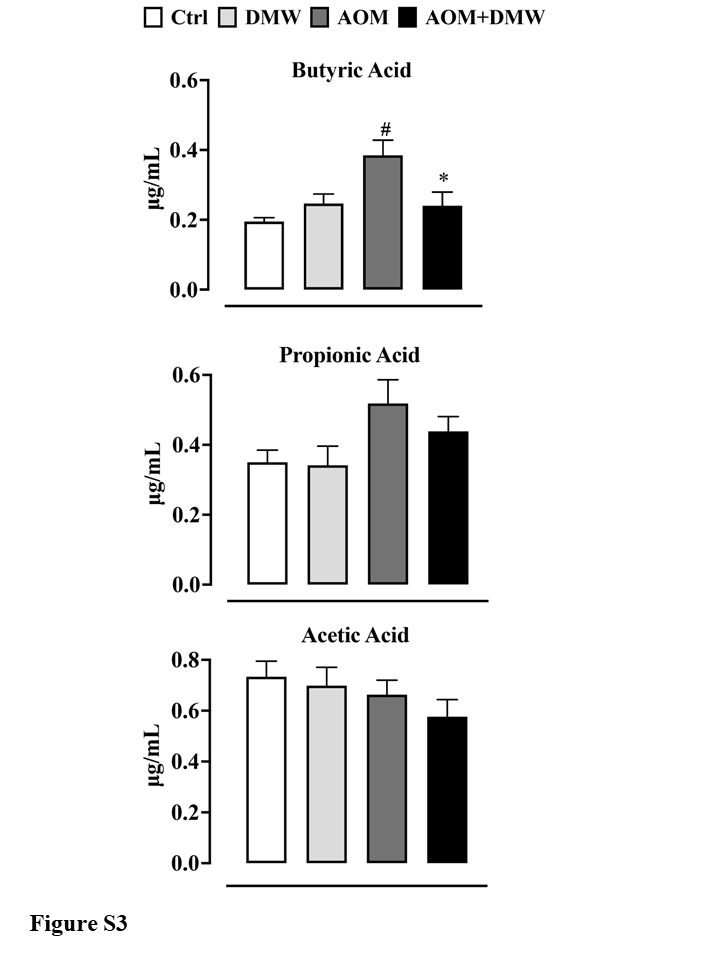

Supplement: Supplementary file 5 — Additional file 4: Supplementary Figure S4. Oral administration of delactosed milk whey by-product (DMW) restores the levels of SCFA affected by azoxymethane (AOM) in serum samples of mice. AOM (40 mg/kg in total, intraperitoneally) was administered, at the single dose of 10 mg/kg, at the beginning of the first, second, third and fourth week. DMW was given (by oral gavage), at the 10 ml/kg dose, three times a week for the whole duration of the experiment starting 1 week before the first administration of AOM. Blood samples were collected by cardiac puncture at the end of experiment (i.e., 13 weeks after the first injection of AOM). Data represent the mean ± SEM of six individual mice sera. #p<0.01 vs control (Ctrl, untreated mice), *p<0.05 vs AOM alone. [file 12964_2023_1271_MOESM4_ESM.tif]

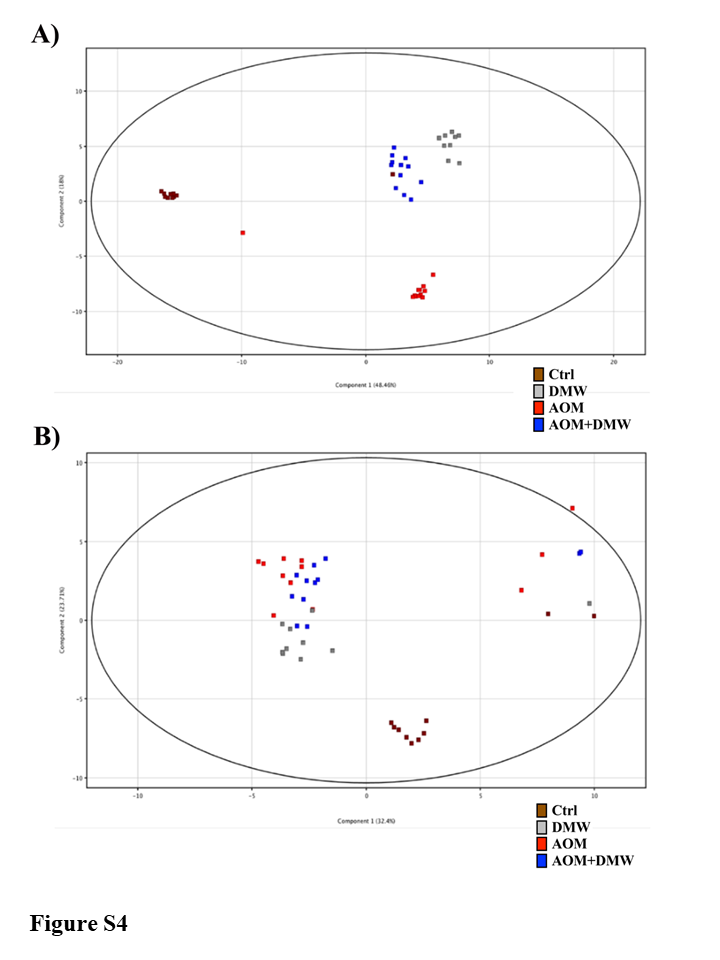

Supplement: Supplementary file 6 — Additional file 5: Supplementary Figure 5. Principal components analysis (PCA) score plots of LC-MS Q-TOF data of water residues of serum after extraction with petroleum ether (A) and ethyl acetate (B). Each group of replicates subjected to different treatments is depicted with a different color: control group (C) = brown; AOM = red; S = grey; AOM added with S = blu. [file 12964_2023_1271_MOESM5_ESM.tif]

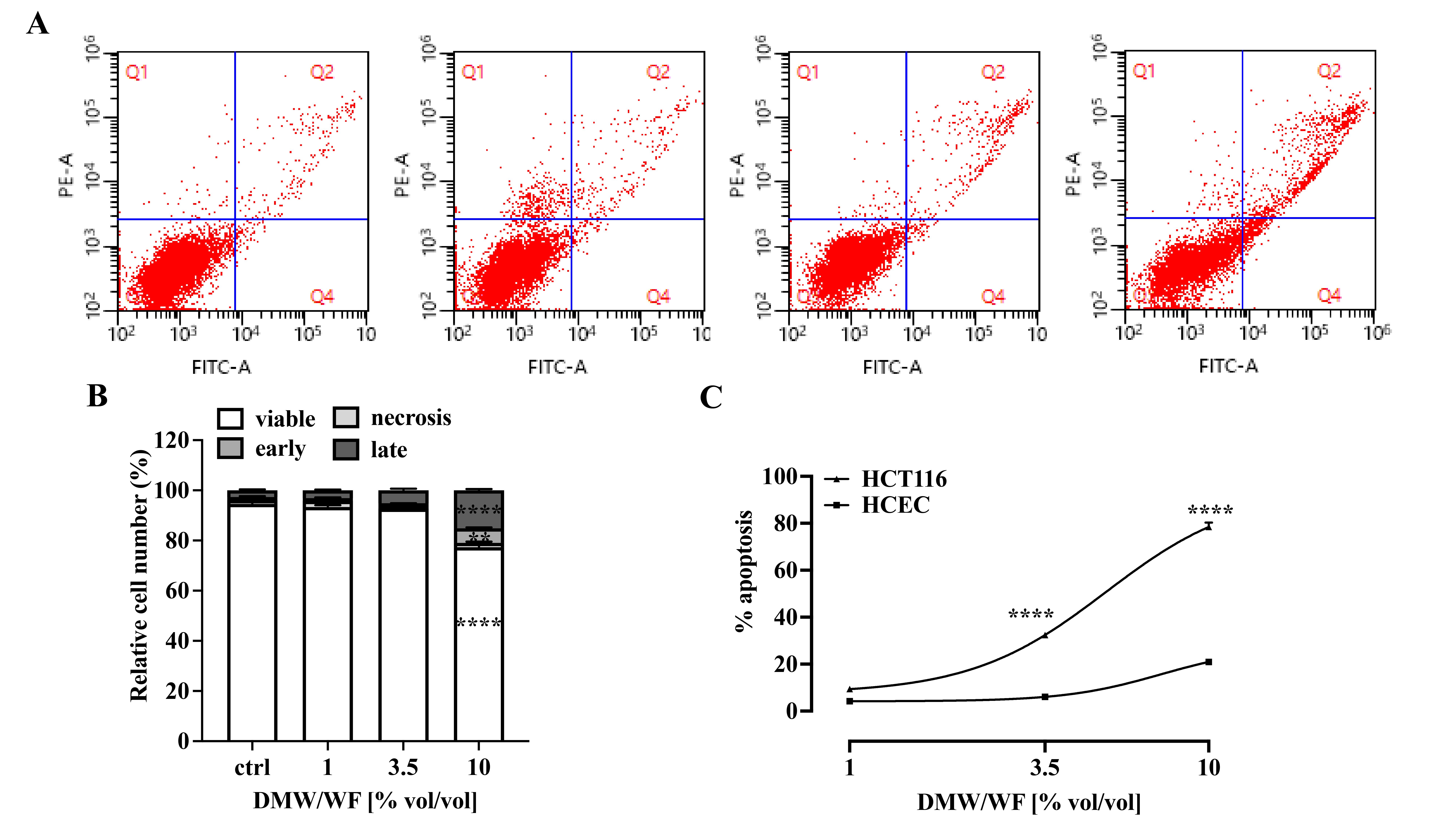

Supplement: Supplementary file 7 — Additional file 6: Supplementary Figure 6. Delactosed milk whey by-product without fat (DMW/WF) causes weak apoptotic effects in HCEC cells. (A-B) HCEC cells were treated with or without increasing concentrations of DMW/WF for 24 hours. Detached and adherent cells were collected and stained with Annexin V and propidium iodide, and then events for early and late apoptotic cells were counted using the BriCyte E6 system (Mindray, PR China) as described in Material and Methods. Data represent the mean ± SEM of two independent experiments. Statistical analysis was performed using ANOVA followed by Dunnett Post-hoc test to determine statistical significance (**p < 0.01; ****p<0.0001 vs ctrl cells). (C) The graph shows the different behavior of HCT116 and HCEC cells in terms of apoptosis induction after exposure to different concentrations of DMW/WF. ****p<0.0001. [file 12964_2023_1271_MOESM6_ESM.tif]
